# Molecular mechanisms of APC/C release from spindle assembly checkpoint inhibition by APC/C SUMOylation

**DOI:** 10.1016/j.celrep.2021.108929

**Published:** 2021-03-30

**Authors:** Stanislau Yatskevich, Jessie S. Kroonen, Claudio Alfieri, Thomas Tischer, Anna C. Howes, Linda Clijsters, Jing Yang, Ziguo Zhang, Kaige Yan, Alfred C.O. Vertegaal, David Barford

**Affiliations:** 1 MRC Laboratory of Molecular Biology, Cambridge Biomedical Campus, Francis Crick Avenue, Cambridge CB2 0QH, UK; 2 Department of Cell and Chemical Biology, Leiden University Medical Center, 2300 RC Leiden, the Netherlands

**Keywords:** APC/C, SUMOylation, SAC, MCC, WHB domain, ubiquitination, cell cycle

## Abstract

The anaphase-promoting complex/cyclosome (APC/C) is an E3 ubiquitin ligase that controls cell cycle transitions. Its regulation by the spindle assembly checkpoint (SAC) is coordinated with the attachment of sister chromatids to the mitotic spindle. APC/C SUMOylation on APC4 ensures timely anaphase onset and chromosome segregation. To understand the structural and functional consequences of APC/C SUMOylation, we reconstituted SUMOylated APC/C for electron cryo-microscopy and biochemical analyses. SUMOylation of the APC/C causes a substantial rearrangement of the WHB domain of APC/C’s cullin subunit (APC2^WHB^). Although APC/C^Cdc20^ SUMOylation results in a modest impact on normal APC/C^Cdc20^ activity, repositioning APC2^WHB^ reduces the affinity of APC/C^Cdc20^ for the mitotic checkpoint complex (MCC), the effector of the SAC. This attenuates MCC-mediated suppression of APC/C^Cdc20^ activity, allowing for more efficient ubiquitination of APC/C^Cdc20^ substrates in the presence of the MCC. Thus, SUMOylation stimulates the reactivation of APC/C^Cdc20^ when the SAC is silenced, contributing to timely anaphase onset.

## Highlights

• APC/C SUMOylation causes substantial rearrangement of the WHB domain of the APC2 subunit
• The repositioned WHB domain prevents robust binding of the MCC to the APC/C
• SUMOylation stimulates robust APC/C reactivation during anaphase onset

## Graphical abstract

**Figure:**
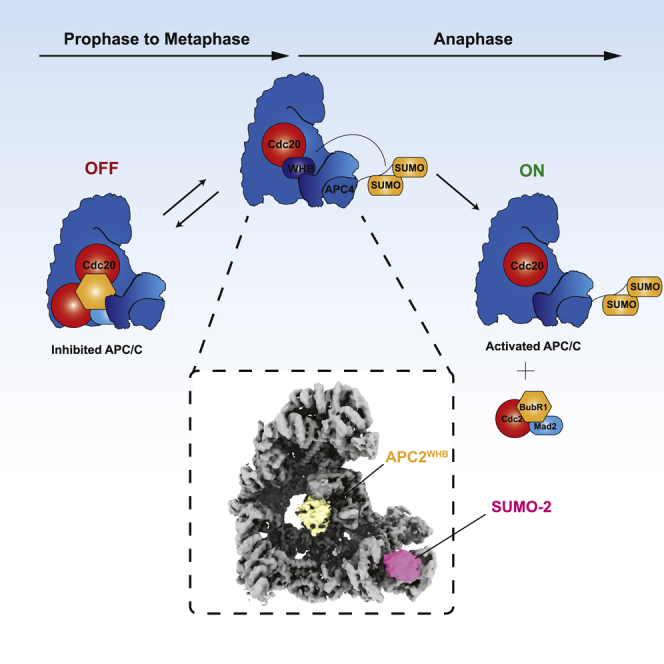

## Teaser

Yatskevich et al. show that SUMOylation of APC/C results in substantial structural rearrangements of the APC/C. These structural changes allow APC/C to be reactivated robustly upon anaphase entry and when spindle assembly checkpoint is silenced, ensuring that mitotic progression is not delayed.

## Introduction

Genetic information must be correctly duplicated and faithfully segregated into two daughter cells to ensure survival of the organism. Correct cell cycle progression depends on reversible protein phosphorylation and irreversible protein degradation. The anaphase-promoting complex/cyclosome (APC/C) is a large E3-ubiquitin ligase that catalyzes formation of K11- and K48-linked poly-ubiquitin chains recognized by the 26S proteasome, thus targeting key substrates for degradation throughout the cell cycle (Alfieri et al., 2017; Watson et al., 2019). Temporal regulation of the APC/C allows the timely degradation of specific substrates. Cyclin A, NEK2A, and HoxC10, for example, are degraded during prometaphase and metaphase (den Elzen and Pines, 2001; Gabellini et al., 2003; Geley et al., 2001; Hayes et al., 2006; Zhang et al., 2019), whereas cyclin B and securin are degraded only after the correct attachment of all chromosomes to a bipolar mitotic spindle, thereby initiating anaphase onset. A specific checkpoint mechanism, the spindle assembly checkpoint (SAC), ensures that the APC/C is inhibited toward cyclin B and securin even by a single improperly attached kinetochore (Musacchio, 2015). The mitotic checkpoint complex (MCC) functions as the effector of the SAC by binding and inhibiting the APC/C bound by its coactivator, Cdc20 (APC/C^Cdc20^). Once all kinetochores have achieved correct attachment to the mitotic spindle, the APC/C is robustly released from spindle checkpoint inhibition within minutes, leading to almost immediate degradation of securin and cyclin B.

The mechanism of APC/C inhibition by the MCC has been defined by cryogenic electron microscopy (cryo-EM) studies of the APC/C-MCC complex (APC/C^MCC^) (Alfieri et al., 2016; Yamaguchi et al., 2016). The APC/C^MCC^ structures showed that the MCC occludes the central cavity of APC/C^Cdc20^. The BubR1 subunit of the MCC occludes substrate recognition sites on both Cdc20 subunits of APC/C^MCC^. In the “closed” state of APC/C^MCC^ (APC/C^MCC-Closed^), BubR1 also binds the WHB domain of APC2 (APC2^WHB^), blocking the catalytic module of APC/C from engaging the priming E2, UbcH10. The APC/C^MCC^ also adopts an “open” conformation in which MCC has swung outward, away from the catalytic module to allow E2 binding.

Even a single unattached kinetochore will delay anaphase onset (Rieder et al., 1995). However, once all kinetochores are attached to microtubules, rapid APC/C reactivation prevents cohesion fatigue that could result in inaccurate sister chromatid segregation, an event that is detrimental to genome integrity. A major discovery was that auto-ubiquitination of MCC components by the APC/C promotes reactivation of the APC/C (Reddy et al., 2007). Cdc20 becomes extensively poly-ubiquitinated in an APC/C-dependent manner (Nilsson et al., 2008). Another candidate for APC/C-dependent ubiquitination of the MCC is BubR1, as its ubiquitination increased significantly when lysine-free Cdc20 was used (Sitry-Shevah et al., 2018), and mutating Cdc20’s two C-terminal ubiquitination sites did not impair MCC dissociation on release from the SAC (Mansfeld et al., 2011). Poly-ubiquitination of Cdc20 of the MCC (Cdc20^M^), and likely BubR1, depends on the APC15 subunit (Alfieri et al., 2016; Yamaguchi et al., 2016). Depletion of APC15 in human cells resulted in increased MCC association with the APC/C, and a pronounced delay of anaphase onset, whereas the normal activity of APC/C *in vitro* was not affected by depleting APC15 (Mansfeld et al., 2011; Uzunova et al., 2012). However, other mechanisms are also likely to be responsible for APC/C^MCC^ disassembly because APC15 deletion in yeast, or depletion in human cells, does not cause a complete arrest at metaphase, in contrast to APC3 depletion (Foster and Morgan, 2012; Mansfeld et al., 2011).

Overall, these data suggest the following mechanism of APC/C reactivation, at least in human cells. The MCC is removed from the APC/C by poly-ubiquitination, and subsequently free MCC is disassembled by the TRIP13-p31^comet^ remodeling complex (Eytan et al., 2014; Habu et al., 2002; Teichner et al., 2011; Xia et al., 2004). This process results in continuous disassembly of the MCC, while unattached kinetochores constantly generate new MCC, imparting responsiveness and robustness to the SAC until all chromosomes are attached. At this point all of the MCC is fully disassembled, and anaphase is initiated.

The small ubiquitin-related modifier (SUMO) family of proteins is frequently attached as a post-translational modification to target proteins to modulate their activity (Gareau and Lima, 2010; Hendriks and Vertegaal, 2016a). Recently, it was discovered that the APC/C is also SUMOylated (Cubeñas-Potts et al., 2015; Eifler et al., 2018; Lee et al., 2018; Matic et al., 2010; Schimmel et al., 2014; Schou et al., 2014). Two conserved lysines of APC4 (K772 and K798) were identified as being SUMOylated. Human cells have four different SUMO isoforms, SUMO1–SUMO4. However, it is unclear whether the SUMO-4 isoform can be properly matured and conjugated to substrates *in vivo* (Owerbach et al., 2005). SUMO-1 shares ∼50% sequence identity with SUMO-2 and SUMO-3, which share 97% sequence identity in their mature forms and are frequently referred to as SUMO-2/3. It was suggested that APC4 was preferentially modified with the SUMO-2/3 isoforms because APC4 is commonly identified in screens for proteins modified with SUMO-2/3. By expressing an APC4 mutant that cannot be SUMOylated, anaphase entry was significantly delayed (Eifler et al., 2018; Lee et al., 2018). Lee et al. (2018) proposed that this delay functions through the SAC based on their finding that reversine, a SAC inhibitor, rescued the delay of anaphase onset in APC4 SUMOylation-defective cells. Eifler et al. (2018) and Lee et al. (2018) also found no effect of SUMOylation on APC/C localization, suggesting an intrinsic mechanism of APC/C regulation.

Herein, we describe the molecular mechanism of APC/C regulation by SUMOylation. We show that SUMOylation rearranges APC2^WHB^ into a position that is incompatible with the MCC binding to APC/C^Cdc20^ in APC/C^MCC-Closed^. We also show that although SUMOylation has a minor effect on APC/C^Cdc20^ ubiquitination activity, it reduces the affinity of APC/C^Cdc20^ for the MCC, attenuating MCC-mediated suppression of APC/C^Cdc20^ activity. This allows for increased cyclin B and securin ubiquitination in the presence of the MCC. We also show that the APC/C is preferentially modified with the SUMO-2 isoform, but that this preference is marginal and results from the shorter flexible N-terminal tail of SUMO-2 compared to SUMO-1. Overall, we present evidence that SUMOylation promotes APC/C reactivation during anaphase by reducing the affinity of APC/C^Cdc20^ for the MCC through rearrangement of APC2^WHB^, thereby destabilizing APC/C^MCC^, and contributing to timely anaphase onset.

## Results

### APC/C is SUMOylated primarily in mitosis

**Figure 1:**
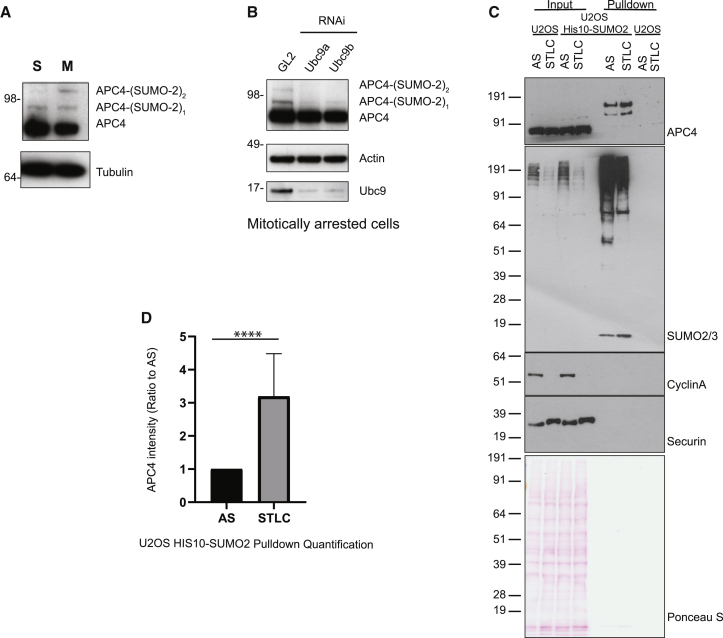
The APC/C is SUMOylated in mitosis (A) APC4 immunoblot of cell lysates (top panel) and tubulin immunoblot of loading control (lower panel). Cells were arrested either in S-phase or in mitosis. The efficiency of the cell cycle arrest was confirmed by immunoblotting against APC3 and phosphorylated histone H3 at Ser10 (Figure S1A). Only mitotically arrested cells show additional SUMOylation bands above APC4. (B) Ubc9 was depleted using 20 nM RNAi. Ubc9 was depleted for 48 h, after which STLC was added to enrich for mitotically arrested cells, and cells were left for an additional 16 h. Immunoblotting against APC4 was performed (top panel). Only the Ubc9-depleted cells (Ubc9a and Ubc9b) showed the disappearance of bands above APC4. RNAi against the luciferase gene (GL2) was used as a control. An anti-Ubc9 immunoblot showed Ubc9 depletion in the Ubc9a- and Ubc9b-treated cells. Actin loading control (middle panel). (C) APC4 SUMOylation is increased in mitotic cells. U2OS HIS10-SUMO2 and U2OS (control) cells, asynchronous (AS) or synchronized in mitosis with STLC, harvested with trypsinization or mitotic shake-off, respectively. Inputs and SUMO2-enriched fractions were analyzed by immunoblotting using antibodies against APC4, SUMO2/3, cyclin A, and securin. Protein loading was verified by Ponceau S staining. The experiment was performed in triplicate and repeats 2 and 3 are shown in Figure S1C. Synchronization was verified by immunoblotting for cyclin A2 and securin and by FACS analysis for DNA content and MPM2 staining and shown in FACS plots (Figure S1D). (D) SUMOylated APC4 from experiments in (C) and its repeats from Figure S1C were quantified and visualized in a graph. Quantification was performed by measuring the band intensity of SUMOylated APC4 in each experiment and comparing it to the intensity of SUMOylated APC4 in AS cells. Data represent the mean with 1 standard deviation (n = 3). Statistical analysis was performed using an unpaired Student’s t test. ^∗∗∗∗^p < 0.001.

A previous study suggested that the APC/C is SUMOylated in a cell cycle-dependent manner (Lee et al., 2018). To unambiguously define at which cell cycle stage the APC/C is SUMOylated, we arrested Flp-In HEK293 T-REx cells either in early S-phase using a double thymidine block, or in prometaphase using the Kif11/Eg5 inhibitor STLC. Cell cycle-specific arrest was confirmed by immunoblotting against both APC3, a protein that is extensively phosphorylated in mitosis, and phosphorylated histone H3, Ser10 (H3S10) (Figure S1A). Cells treated with STLC showed a phosphorylated APC3 band and had a signal for H3S10 phosphorylation, confirming efficient arrest at mitosis. Blotting for APC4 revealed that two additional bands, above the main APC4 band, were present specifically in mitotically arrested cells. A single additional band was observed in S-phase-arrested cells (Figure 1A). To confirm that these bands represented SUMOylated APC/C, we depleted Ubc9, the sole E2 enzyme of the SUMOylation pathway. In mitotically arrested cells, Ubc9 depletion resulted in the disappearance of the two bands above APC4, suggesting that these two bands are mono- and di-SUMOylated APC4 (Figure 1B). Depletion of Ubc9 in cycling cells resulted in the disappearance of the single band above the APC4 band (Figure S1B).

To further confirm that the two additional bands above APC4 are SUMOylation bands, and to quantify the increase in APC4 SUMOylation during mitosis, U2OS His-SUMO-2 and U2OS (control) cells were synchronized in mitosis using the Eg5 inhibitor STLC, and a His-SUMO-2 pull-down was performed (Figure 1C; Figure S1C). Cell cycle synchronization in these experiments was verified by immunoblotting for cyclin A2 and securin (Figure 1C), and by fluorescence-activated cell sorting (FACS) analysis for DNA content and MPM2 staining (Figure S1D). These experiments showed that there was a 3-fold enrichment of APC4 SUMOylation in STLC-arrested cells compared to asynchronous cells (Figure 1D).

We also tested whether APC/C SUMOylation depends on any of the known E3 SUMO ligases. The only E3 SUMO ligase that we could reliably deplete was RanBP2 (Figure S1E). Interestingly, we observed that, relative to control cells, APC4 SUMOylation was abolished when either Ubc9 or RanBP2 was depleted.

Overall, our data show that the APC/C is preferentially SUMOylated in mitosis and that the fraction of SUMOylated APC/C in cells is low. Our data also suggest that APC4 SUMOylation depends on RanBP2. Interestingly, RanBP2 is located at the kinetochore in mitotic cells, which is also where APC/C can be found during mitosis. However, we cannot exclude the possibility that the effect of RanBP2 knockdown on APC4 SUMOylation is indirect.

### The APC/C is SUMOylated preferentially by the SUMO-2 isoform due to its shorter N-terminal domain

To reconstitute APC/C SUMOylation *in vitro* to better dissect the mechanism of APC/C SUMOylation and the function of SUMOylated APC/C, we purified the E1 and E2 enzymes of the SUMOylation pathway and different isoforms of SUMO. First, we determined whether our *in vitro* system recapitulated the APC/C SUMOylation observed *in vivo*, in which SUMO-2/3 was detected on APC4 as mono-attachments at lysines 772 and 798 (Cubeñas-Potts et al., 2015; Eifler et al., 2018; Lee et al., 2018; Matic et al., 2010). We performed SUMOylation reactions using unmodified SUMO-2 and a SUMO-2 mutant in which all lysines were mutated to arginines (SUMO-2^KR^) (Figure S2A). SUMO-2^KR^ modifies single lysines, but it cannot form chains. We detected mono- and di-SUMO modifications of APC4 in the presence of both SUMO-2 and SUMO-2^KR^, indicating that our *in vitro* system modifies two sites on APC4 by addition of a single SUMO moiety. To further test whether these two SUMO-2 moieties were either attached to individual lysines or formed a SUMO chain, we treated our SUMOylated APC/C with SENP2 SUMO-protease (Reverter and Lima, 2004). Blotting against APC4 and SUMO-2/3 showed that our SUMOylated APC/C contains almost exclusively di-SUMOylated APC4 (Figure S2B). Additionally, SENP2, a SUMO protease that acts on terminal SUMO moieties, removed SUMO-2 from the *in vitro* reconstituted SUMOylated APC/C, although SENP1, and not SENP2, was proposed to remove SUMO from APC/C *in vivo* (Lee et al., 2018). Thus, these experiments demonstrated that recombinantly produced and purified APC/C is SUMOylated *in vitro* on two lysines on APC4, mimicking the APC/C SUMOylation state *in vivo*.

**Figure 2:**
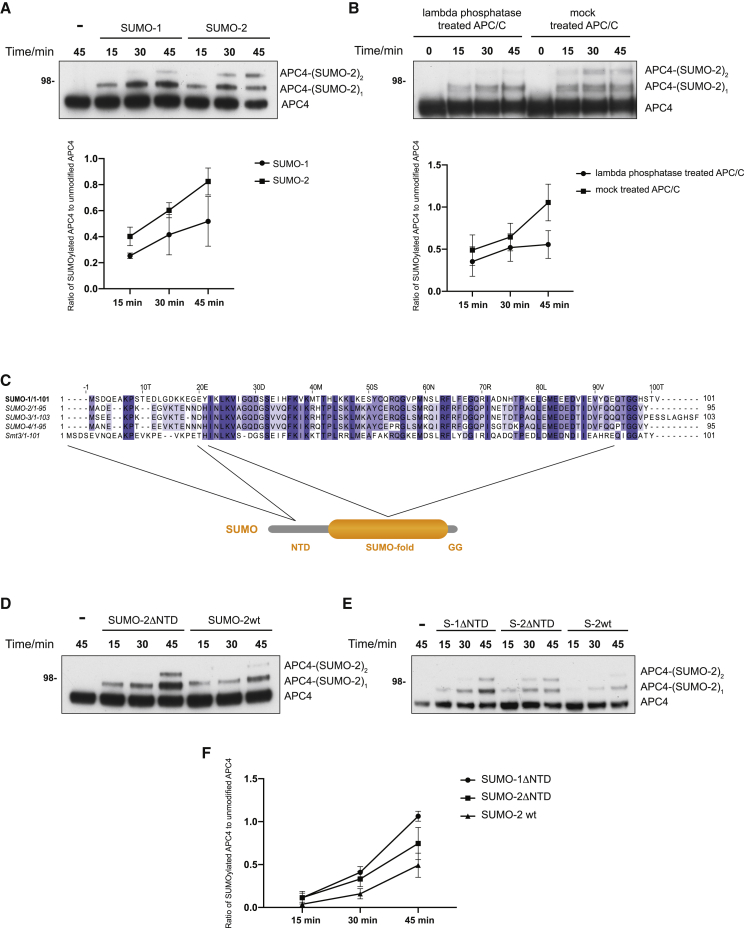
The APC/C is preferentially SUMOylated *in vitro* by the SUMO-2 isoform due to its shorter N-terminal domain (A) A SUMOylation assay showed that APC/C SUMOylation by SUMO-2 is faster than by SUMO-1. Recombinantly expressed and purified APC/C was used as a substrate in the presence of Uba2-Aos1 (E1 enzymes), Ubc9, and either SUMO-1 or SUMO-2. The SUMOylation reaction was analyzed by immunoblotting with an anti-APC4 antibody to detect APC4 SUMOylation. The assay was quantified as described in STAR Methods, and results are plotted in the lower panel. For each time point, the ratio of modified APC4 to unmodified APC4 was calculated and plotted as mean with standard deviation for each time point. The assay was performed in triplicate. (B) Partially phosphorylated APC/C is SUMOylated faster than non-phosphorylated APC/C. To obtain non-phosphorylated APC/C, purified APC/C was treated with lambda phosphatase for 30 min, after which the SUMOylation reaction mix was added and the SUMOylation assay was performed as described in STAR Methods and above. As a control, mock-treated APC/C was incubated with a lambda phosphatase buffer. The assay was quantified as described in STAR Methods, and results are plotted on the lower panel. For each time point, the ratio of modified APC4 to unmodified APC4 was calculated and plotted as mean with standard deviation for each time point. The assay was performed in triplicate. (C) Multiple sequence alignment of SUMO isoforms shows that the N-terminal domain (NTD) is the most sequence-divergent region. (D) The APC/C is SUMOylated faster by SUMO-2 lacking its NTD (SUMO-2ΔNTD). The SUMOylation reaction was performed as described in STAR Methods and above. SUMO-2 variants loading was confirmed by a separate SDS-PAGE gel stained with Coomassie blue, where SUMO-2 and SUMO-2ΔNTD were loaded at the same amounts used in this assay (Figure S2I). (E) The rate of APC/C SUMOylation by SUMO-1 and SUMO-2 isoforms is identical when both proteins lack their NTD. Both the SUMO-1 and SUMO-2 isoforms without their NTDs (S-1ΔNTD and S-2ΔNTD, respectively) resulted in the same rate of APC/C SUMOylation. This was faster than the rate of APC/C SUMOylation with unmodified wild-type SUMO-2. (F) Quantification of assay in Figure 2E. The assays were quantified as described in STAR Methods, and results are plotted on the lower panel. For each time point, the ratio of modified APC4 to unmodified APC4 was calculated and plotted as mean with standard deviation for each time point. The assay was performed in triplicate.

Previous studies detected SUMO-2 modification of APC/C *in vivo* (Cubeñas-Potts et al., 2015; Eifler et al., 2018; Lee et al., 2018; Matic et al., 2010). To better understand the mechanism of APC/C SUMOylation, and what factors contribute to it, we performed SUMOylation reactions using SUMO-1 and SUMO-2. Modification with SUMO-2 proceeded faster than with SUMO-1 (Figure 2A; Figure S2C). Previous studies identified APC4 SUMOylation by pull-down of N-terminally His-tagged SUMO-2 (Matic et al., 2010). When we performed our SUMOylation assay using His-tagged SUMO-1 and His-tagged SUMO-2 (Figures S2D and S2E), the His-tagged SUMO-2 isoform SUMOylated APC/C considerably faster than His-tagged SUMO-1, suggesting that N-terminally tagged SUMO isoforms might bias the prevalence of one SUMO isoform over others for certain proteins. We next directly compared the rate of APC/C SUMOylation using unmodified and His-tagged SUMO-2 (Figure S2F and Figure S2G). This showed that APC/C is modified much more efficiently by unmodified SUMO-2, confirming that the His-tag interferes with APC/C SUMOylation.

Previous studies had shown that the APC/C with APC4 phosphorylated at S777 and S779 is a preferred substrate for Ubc9 (Eifler et al., 2018). Mass spectrometry showed that our purified recombinant APC/C is phosphorylated at S777 of APC4, and we also sometimes detect phosphorylation on S779. Treatment with lambda phosphatase completely dephosphorylated these sites (Figure S2H). We performed the SUMOylation reaction using dephosphorylated or mock-treated APC/C and observed that mock-treated APC/C is SUMOylated more efficiently than dephosphorylated APC/C (Figure 2B). This confirms that APC/C SUMOylation is stimulated by phosphorylation, as observed previously *in vivo* (Eifler et al., 2018).

We then determined what causes the slight preference for SUMO-2 *in vitro* compared to SUMO-1. Multiple sequence alignment of SUMO isoforms (Figure 2C) shows that the N-terminal domain (NTD) of SUMO is the most sequence-divergent region. We reasoned that isoform specificity might be governed by differences in this NTD. To test this, we generated SUMO-2 with its NTD deleted (SUMO-2^ΔNTD^). Interestingly, the SUMOylation reaction proceeded even faster with SUMO-2^ΔNTD^ (Figure 2D; Figure S2I). Together with our observation that an N-terminal His tag interferes with the SUMOylation reaction, this suggested that the length of the NTD might determine SUMO isoform specificity for APC/C, given that the NTD of SUMO-2 is four amino acids shorter than that of SUMO-1. To test this, we prepared SUMO-1 that lacks an NTD (SUMO-1^ΔNTD^) and thus has an identical length to SUMO-2^ΔNTD^ at its N terminus. Interestingly, the rate of APC/C SUMOylation was now even faster for SUMO-1^ΔNTD^ compared to SUMO-2^ΔNTD^, and both of these reactions were faster still than full-length SUMO-2 (Figures 2E and 2F; Figure S2J). Thus, the preference for wild-type SUMO-2 is marginal, and SUMO^NTD^ plays an important role in conferring this specificity.

### APC/C SUMOylation promotes repositioning of APC2^WHB^

**Figure 3:**
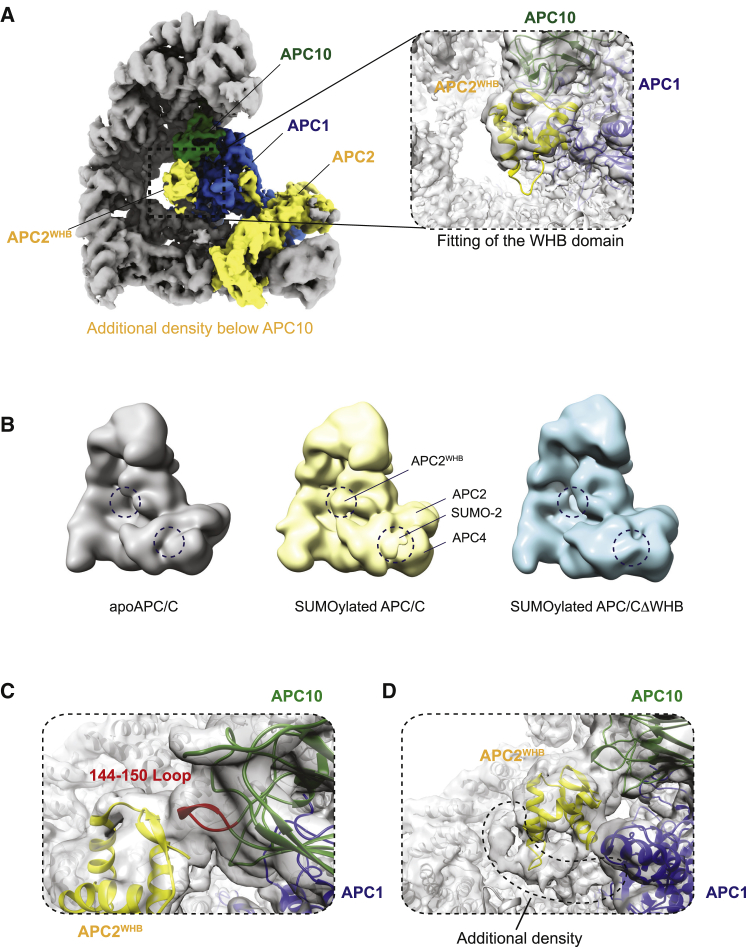
APC/C SUMOylation results in repositioning of APC2^WHB^ below APC10 (A) Cryo-EM map of SUMOylated APC/C. APC10 (forest green), APC1 (blue), APC2 (yellow). APC2^WHB^ is positioned below and in contact with APC10. The dashed insert box highlights the interactions of the repositioned APC2^WHB^ in contact with APC10. The EM density map is shown as a white transparent surface, and APC1, APC10, and APC2^WHB^ are shown in cartoon representations. (B) A comparison of the non-SUMOylated APC/C, SUMOylated APC/C, and SUMOylated APC/C^ΔWHB^ cryo-EM maps low pass filtered to 20 Å resolution shows that the APC2^WHB^ density (dashed circle) is present in the SUMOylated APC/C structures, but not in non-SUMOylated APC/C. (C) APC2^WHB^ interacts with the amino acids 144–150 of APC10 (144–150 loop, in red). EM map is shown as a white transparent surface. (D) At a lower EM density threshold, additional continuous density is observed below APC2^WHB^ (indicated by the dashed line). This continuous density is located between APC2^WHB^ and APC11.

To address the structural and functional consequences of APC/C SUMOylation, we prepared (SUMO-2)_2_-APC/C by *in vitro* SUMOylation. As judged by SDS-PAGE and immunoblotting, we obtained near stoichiometric modification of APC4 with two SUMO-2 moieties (Figures S2B and S3A). We prepared cryo-EM grids and determined the structure of the SUMOylated APC/C (Figure 3A; Figures S3B–S3E; Table S1). This revealed additional EM density situated within the central cavity, attached to APC10. Focused 3D classification around this region, followed by multi-body refinement (Zivanov et al., 2018), resulted in significantly improved EM density (Figure S3F). Interestingly, this additional density did not match a SUMO domain, but instead could easily fit APC2^WHB^, a highly mobile domain at the C terminus of APC2 that is not normally visible in apoAPC/C structures (Chang et al., 2015, Brown et al., 2015). The estimated local resolution in this region of the map is between 5 and 7 Å, and we could readily trace the polypeptide backbone of the APC2^WHB^ domain (Figure S3E). To further verify that this additional density is APC2^WHB^, we generated an APC/C mutant with APC2^WHB^ deleted (APC/C^ΔWHB^) and determined a cryo-EM structure of SUMOylated APC/C^ΔWHB^ (Figure 3B; Figure S4A). We also reprocessed original APC/C cryo-EM datasets to ensure that this extra density had not escaped prior notice (Zhang et al., 2016). No additional density was present for both the non-SUMOylated apoAPC/C and SUMOylated APC/C^ΔWHB^ (Figure 3B), confirming this density as APC2^WHB^ resulting from APC2^WHB^ repositioning due to APC/C SUMOylation. Interestingly, in a previous study (Brown et al., 2014), similar EM density below APC10 was observed for the APC/C in complex with Ube2S.

APC2^WHB^ binds directly to a loop of APC10 comprising residues 144–150 (Figure 3C; Figure S4B). This loop does not participate extensively in substrate binding (Chang et al., 2015). Thus, the repositioned APC2^WHB^ would marginally clash with coactivator-bound structures (Figure S4C). At a lower EM density threshold, we also observed an additional continuous density below APC2^WHB^ (Figure 3D), which we cannot clearly assign, but which makes interactions with APC2^WHB^, APC1, and APC10, stabilizing APC2^WHB^ in this position. This density is continuous and consistent with being an extended polypeptide chain. This additional density could be part of one of the SUMO-2 moieties, such as the SUMO-2 N-terminal extension, potentially from the second SUMO-2, that additionally stabilizes the APC2^WHB^ at this position.

### SUMO-2 binds to the APC2–APC4 subunit interface

**Figure 4:**
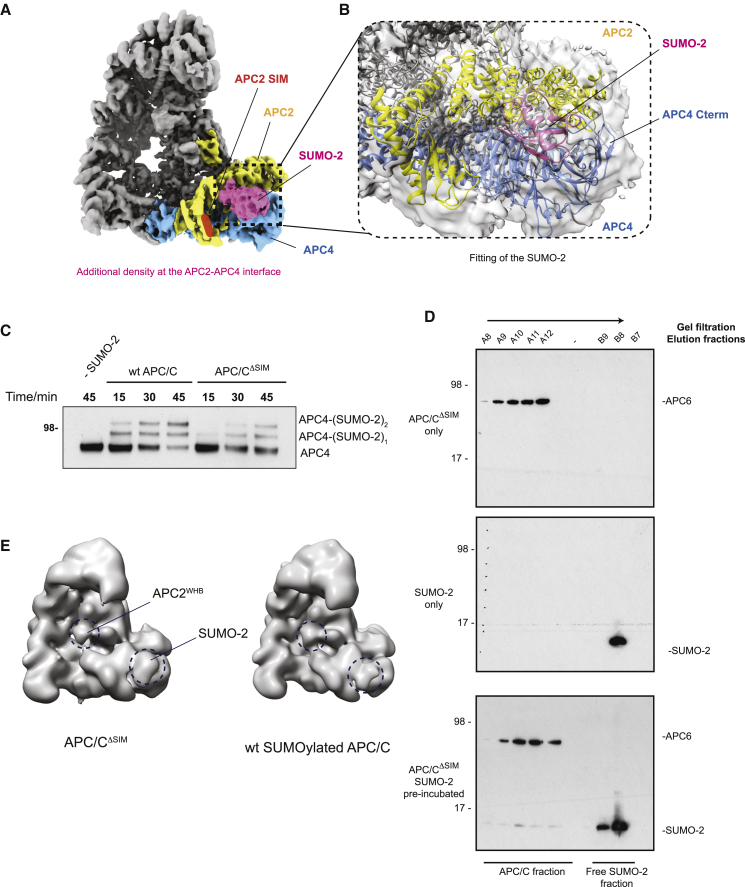
SUMO-2 binds directly to APC/C, independent of the APC2 SIM (A) Cryo-EM map of a separate 3D class of SUMOylated APC/C shows a strong density feature (pink) at the APC2–APC4 interface. No connection between this density and the SIM on APC2 (indicated by a red ellipse) can be observed. Light blue indicates APC4; yellow indicates APC2; pink indicates new additional density. (B) A model of SUMO-2 fits well to this additional density at the APC2–APC4 interface (SUMO-2 PDB: 1WM3). (C) APC/C with the SIM mutated (APC/C^ΔSIM^) is SUMOylated at a reduced rate compared to wild-type APC/C (WT APC/C). SUMOylation assays were performed as described in STAR Methods and Figure 2. (D) Analytical gel filtration reconstitution experiments show that SUMO-2 binds to APC/C^ΔSIM^. APC/C^ΔSIM^ at 1 μM was incubated with 60 μM SUMO-2 on ice for 30 min and then samples were injected onto a Superose 6 gel filtration column. The chromatogram is shown in Figure S5C. The samples were analyzed by immunoblotting against SUMO-2 and APC6. (E) Comparison of the cryo-EM maps of SUMOylated APC/C^ΔSIM^ and SUMOylated wild-type APC/C structures low pass filtered to 20 Å resolution shows that mutating the SIM on APC2 has no effect on the structure of SUMOylated APC/C. The positions of APC2^WHB^ and SUMO-2 are indicated by dashed circles.

The EM data of SUMOylated APC/C also revealed an additional density feature lying immediately above APC4 and adjacent to APC2 that was absent in our reprocessed unmodified apoAPC/C datasets (Figure 3B; Figure S4D). We performed focused 3D classification around this APC2–APC4 region to increase occupancy around that site and to improve the resolution (Figure S4E). The density was highly variable, as shown by 3D classification where we observed a small density feature distributed along the entire surface of the WD40 domain of APC4, making it difficult to isolate a single class at high resolution. The best EM density map had this additional density contacting both the APC2 cullin repeats and the WD40 domain of APC4 (Figure 4A; Figure S4E). Since this density is exclusive to the SUMOylated APC/C structure, and appears to interact with APC4, we reasoned that it might correspond to SUMO-2. This density is also present in our map of SUMOylated APC/C^ΔWHB^, indicating that it cannot be APC2^WHB^ (Figure 3B). This additional density feature closely matches the SUMO-2 atomic model (Figure 4B). Interestingly, this would potentially position the C-terminal GG motif of SUMO-2 (the attachment site for substrates) outward near APC4’s C-terminal disordered region, where SUMO is attached to the APC/C (Figure 4B). Additionally, the N terminus of SUMO-2 would be facing the APC2–APC4 interface and, if the SUMO-2^NTD^ was too long, it would clash with APC2–APC4, disrupting APC/C-SUMO interactions. This might explain why the length of the SUMO-2^NTD^ is crucial for efficient APC/C SUMOylation. Lastly, the density could only fit a single SUMO-2 molecule. Although our *in vitro* SUMOylated APC/C sample has two SUMO-2 molecules covalently linked to APC4, no obvious additional density for a second simultaneous SUMO-2 moiety is visible in our maps.

Our model suggests direct interactions between SUMO-2 and the APC/C. To test this, we performed size exclusion chromatography with free APC/C and SUMO-2, and the pre-incubated APC/C-SUMO-2 complex. SUMO-2 eluted earlier in APC/C-containing fractions when it was pre-incubated with the APC/C, suggesting it binds non-covalently to the APC/C (Figures S5A and S5B). Previously, Lee et al. (2018) identified a SUMO interaction motif (SIM) on APC2 (residues 727–732, Figure 4A). They showed that *in vitro*-translated APC2 binds SUMO-2, and that mutating two buried residues, V728 and L729, to Ala abolished APC2 interaction with SUMO-2 (Lee et al., 2018). However, we do not observe EM density connecting APC2 SIM and SUMO-2 in our structure, nor do we see any additional EM density associated with the APC2 SIM. Therefore, we tested whether SUMO-2 has an additional interaction mechanism with APC/C that is independent of APC2’s SIM. We disrupted the SIM in the APC/C by mutating V728, L729, and I730 of APC2 to Ala (APC/C^ΔSIM^). We observed that the rate of APC/C^ΔSIM^ SUMOylation was significantly reduced compared to wild-type APC/C (Figure 4C). As assessed by size exclusion chromatography, we found that SUMO-2 still bound APC/C^ΔSIM^, suggesting that the APC2 SIM is not necessary for SUMO-2 binding to the APC/C (Figure 4D; Figure S5C). This is consistent with our structure, where SUMO-2 interacts with both APC2 and APC4. However, our interaction assay between APC/C and free SUMO-2 is only qualitative, so we cannot exclude the possibility that APC2^SIM^ contributes to the SUMO-2 affinity for the APC/C. It is also possible that the second SUMO-2 moiety interacts with the APC2^SIM^ and this interaction is simply not resolved in our structural work, but it is nevertheless important for APC/C SUMOylation. Lastly, we determined whether APC/C^ΔSIM^ undergoes the same structural rearrangements as wild-type protein as a result of SUMOylation. The cryo-EM structure of SUMOylated APC/C^ΔSIM^ is virtually indistinguishable from the wild-type SUMOylated APC/C (Figure 4E). We confirmed that our purified APC/C^ΔSIM^ had the identical composition to wild-type protein and that it was properly SUMOylated (Figure S5D).

In a structure of the APC/C-NEK2A complex (Alfieri et al., 2020), APC2^WHB^ is repositioned to the APC2–APC4 interface, similar to where we assign the SUMO-2 density (Figure S5E). In addition, our re-analysis of the APC/C in complex with the TAME inhibitor (an IR-tail mimic) (Zhang et al., 2016) also shows density for APC2^WHB^ at this site. We tested whether the interaction of SUMO-2 at the APC2–APC4 interface is important for APC/C SUMOylation by comparing the SUMOylation rates of wild-type APC/C and the APC/C^ΔWHB^ mutant. The APC/C^ΔWHB^ mutant was SUMOylated marginally faster than the wild-type APC/C (Figure S5F). This is presumably because APC2^WHB^, which sometimes binds at the APC2–APC4 interface, is removed and no longer competes with SUMO-2 for the same binding site.

Lastly, apart from the repositioned APC2^WHB^ domain and new density at the APC2–APC4 interface, the other differences are small rearrangements of the APC2 subunit, as can be seen in the difference map between apoAPC/C and SUMOylated APC/C (Figure S4D). This suggests conformational changes or rigidification of APC2 cullin repeats and APC2^CTD^ in response to SUMOylation.

We conclude that SUMO-2 binds directly to the APC/C, with the major binding site formed from the APC2–APC4 interface, as well as contributions from the SIM of APC2. We observe that although the APC2 SIM is important for optimal APC/C SUMOylation, it is dispensable for the relocation of APC2^WHB^ to APC10.

### SUMOylation has little effect on APC/C^Cdc20^ activity

**Figure 5:**
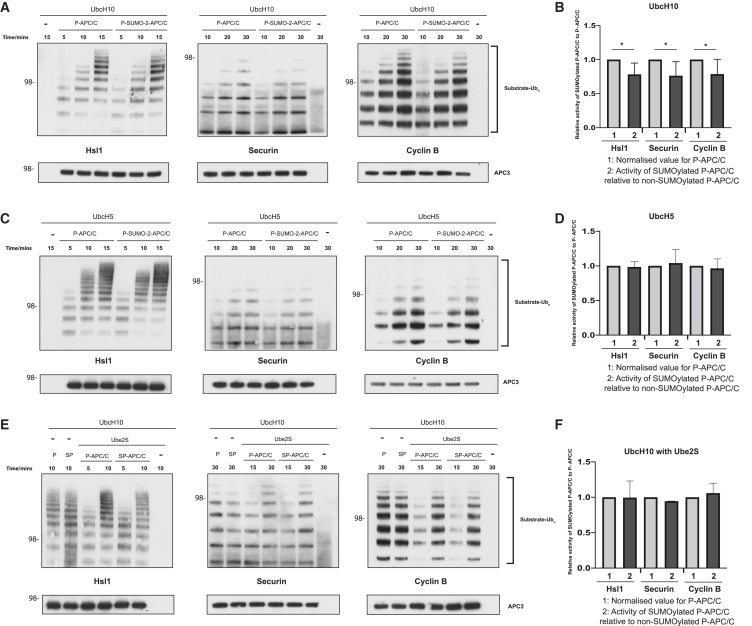
SUMOylation has a marginal effect on APC/C^Cdc20^ ubiquitination activity (A) Comparison of SUMOylated and non-SUMOylated APC/C ubiquitination activity toward three substrates using UbcH10 as a sole E2 enzyme. The ubiquitination assay was performed as described in STAR Methods. 15 μM CDK1/2 III inhibitor was also added to reactions that contained cyclin B. The reactions proceeded for the time indicated in the figure. Reactions using Hsl1 and cyclin B as substrates were analyzed by immunoblotting against the His-tag on ubiquitin, whereas reactions using securin were analyzed by immunoblotting against securin. The APC/C loading control was performed by immunoblotting against APC3 (lower panels), which also confirms that APC/C was phosphorylated equally well. APC/C samples used for all of these assays were also compared on SDS-PAGE gels stained with Coomassie blue (Figure S6A). All experiments were performed at least in triplicate. (B) Quantification of (A). The light gray bar is the normalized activity of non-SUMOylated APC/C, set to 1, and the adjacent black bar is the ratio of the SUMOylated/non-SUMOylated APC/C activity. Standard deviations were calculated for these ratios and account for difference in both samples. Quantification was performed as described in STAR Methods by comparing the lanes of SUMOylated and non-SUMOylated APC/C at identical time points. Data represent the mean with one standard deviation ^∗^p < 0.05. Significance was calculated using the unpaired Student’s t test. (C) Comparison of SUMOylated and non-SUMOylated APC/C ubiquitination activity toward three substrates using UbcH5 as a sole E2 enzyme. Ubiquitination assays were performed as in STAR Methods with a difference that 300 nM UbcH5 was used instead of UbcH10. All experiments were performed at least in triplicate. (D) Quantification of (C), as described in (B) and STAR Methods. No statistically significant difference was found between the samples. (E) Comparison of SUMOylated and non-SUMOylated APC/C ubiquitination activity toward three substrates using UbcH10 supplemented with Ube2S. Ubiquitination assays were performed as described in STAR Methods with a difference that 150 nM UbcH10 was supplemented with 300 nM Ube2S. All experiments were performed at least in triplicate. (F) Quantification of (E), as described in (B) and STAR Methods. No statistically significant difference was found between the samples.

To understand the functional consequences of the structural rearrangements we observed, we performed ubiquitination assays *in vitro* using Cdc20 as the coactivator and either phosphorylated APC/C (p-APC/C) or phosphorylated and SUMOylated APC/C (p-SUMO-2-APC/C, Figure S6A). First, we used the canonical APC/C priming E2 UbcH10, in the absence of Ube2S. We ensured that our assay was in the linear range by titrating UbcH10 into the ubiquitination reaction (Figure S6B). The processivity of SUMOylated APC/C was reduced compared to that of non-SUMOylated APC/C (Figures 5A and 5B). The effect was modest but present for all substrates tested, that is, the model substrate Hsl1, securin, and cyclin B. UbcH10 activity absolutely requires APC2^WHB^ (Brown et al., 2015), and we reasoned that since APC2^WHB^ of SUMOylated APC/C is repositioned, then the APC/C might have a reduced affinity for UbcH10, explaining the reduced ubiquitination processivity we observed. To test this hypothesis, we examined another E2 enzyme, UbcH5, important for APC/C function (Wild et al., 2016), but that does not require APC2^WHB^ (Brown et al., 2015). We also ensured that the assay with UbcH5 was in the linear range (Figure S6B). In this instance, the activities of both SUMOylated and non-SUMOylated APC/C were identical (Figures 5C and 5D), showing that the difference in activity with UbcH10 was caused by its dependence on APC2^WHB^, consistent with our structural results. This result indicates that the repositioned APC2^WHB^ in SUMOylated APC/C does not interfere with binding of the three D-box-containing substrates. Finally, we tested whether APC/C activity was affected when UbcH10 was supplemented with the elongating E2, Ube2S (Brown et al., 2016). Ube2S restored the decreased APC/C processivity with UbcH10 alone. There was no difference between SUMOylated and non-SUMOylated APC/C (Figures 5E and 5F). A previous study suggested that Kif18B, an APC/C substrate, was preferentially ubiquitinated by SUMOylated APC/C, using a different experimental approach that was expected to co-purify endogenous KIF2C together with exogenous Kif18B (Eifler et al., 2018). We performed ubiquitination assays with purified Kif18B in the absence of KIF2C. Kif18B by itself was a poor substrate *in vitro*, but that, similar to other substrates we tested, was not more efficiently ubiquitinated by SUMOylated APC/C (Figure S6C).

Lastly, we tested whether SUMOylated APC/C^ΔSIM^ behaved similarly to wild-type SUMOylated APC/C since the same structural rearrangements occur for both complexes. We found that phosphorylated APC/C^ΔSIM^ had the same activity as phosphorylated wild-type APC/C (Figure S6D), indicating that the ΔSIM mutation does not influence APC/C activity. Also similar to wild-type SUMOylated APC/C, SUMOylated APC/C^ΔSIM^ had a reduced response to UbcH10 (Figure S6E), consistent with their equivalent structures.

Overall, these results reveal that SUMOylation has a limited effect on APC/C ubiquitination activity toward different substrates *in vitro*. Furthermore, although the APC2 SIM is required for optimal rates of APC/C SUMOylation, once SUMOylated, APC2 SIM does not influence APC/C activity.

### SUMOylation reduces APC/C^Cdc20^ affinity for the MCC

**Figure 6:**
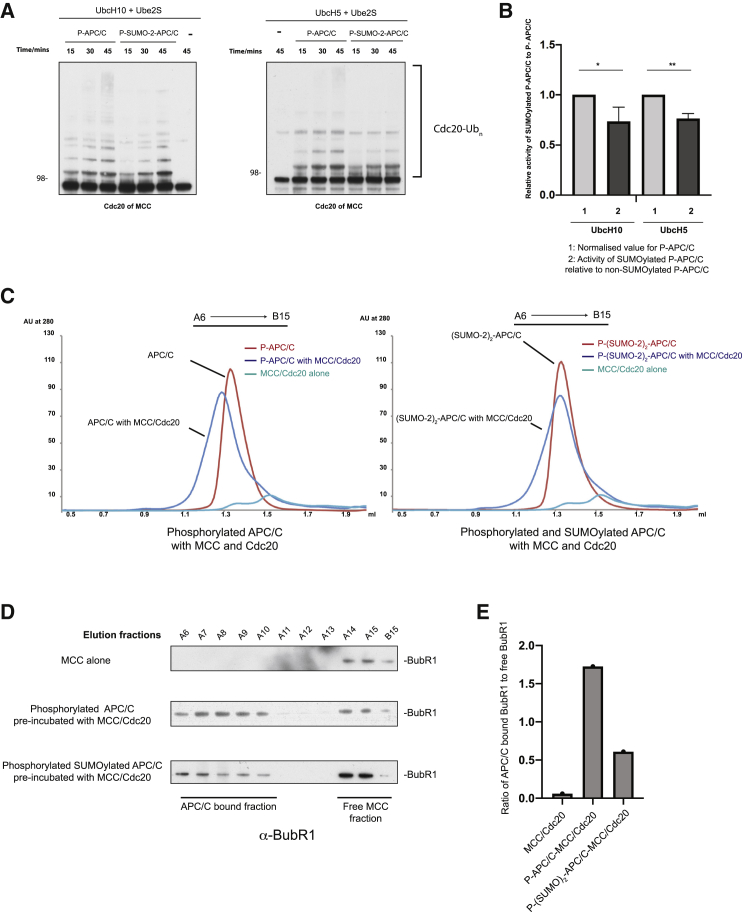
SUMOylated APC/C has reduced affinity for MCC (A) A ubiquitination assay that compares SUMOylated and non-SUMOylated APC/C auto-ubiquitination activity toward Cdc20 of MCC. Ubiquitination reactions were performed similarly to Figure 5 except for using 200 nM recombinant APC/C, 200 nM recombinant human Cdc20, 200 nM recombinant human MCC, and 300 nM Ube2S in the presence of either 300 nM UbcH5 or UbcH10. Cdc20 auto-ubiquitination was detected by immunoblotting against Cdc20. (B) Quantification of (A), as described in Figure 5B and STAR Methods. (C) Analytical gel filtration showed that SUMOylated APC/C binds less well to MCC compared to non-SUMOylated APC/C. Non-SUMOylated phosphorylated APC/C and SUMOylated phosphorylated APC/C at 1 μM were pre-incubated on ice for 30 min together with 1.5 μM MCC and 1.5 μM additional Cdc20. The samples were separated on a Superose 6 gel filtration column, and their elution profiles without any adjustments are shown. Free MCC was included as a control. (D) Immunoblotting against BubR1 of all peak fractions from APC/C and MCC/Cdc20 reconstitution. BubR1 was found in the early fractions only in the presence of APC/C. The fractions taken for the immunoblotting are shown in (C). (E) Quantification of (D). To quantify the results, the intensity of BubR1 bands was measured using ImageJ of APC/C-bound fractions and free MCC fractions separately. The ratio was calculated by dividing total intensity of BubR1 in A6–A10 lanes of APC/C-bound fractions by the A14–B15 intensity of free MCC fractions.

Our structure indicated that the repositioned APC2^WHB^, promoted by APC/C SUMOylation, is incompatible with APC/C^MCC-Closed^ (Alfieri et al., 2016; Yamaguchi et al., 2016). This is because on MCC binding, Cdc20^A^ of APC/C^Cdc20^ is tilted and shifted to occupy a position closer to APC2, which would sterically clash with APC2^WHB^ in the SUMOylated APC/C structure (Figure S7A) (Qiao et al., 2016; Zhang et al., 2016). Additionally, BubR1 of the MCC also engages the 144–150 loop of APC10, and APC2^WHB^ would directly compete with BubR1 for this binding site (Figure S7B). However, the repositioned APC2^WHB^ has less steric collisions with APC/C^MCC-Open^ (Figure S7C). To test the possibility that the affinity of APC/C^Cdc20^ for the MCC is reduced as a result of APC/C SUMOylation, we assessed the rate of auto-ubiquitination of the MCC Cdc20 subunit (Cdc20^M^). This showed that auto-ubiquitination of Cdc20^M^ was reduced using both E2s, UbcH10 and UbcH5, even in the presence of Ube2S (Figures 6A and 6B). This contrasts with the lack of effect we observed for substrates in the absence of the MCC (Figures 5E and 5F), thus supporting the idea that SUMOylation influences the stability of APC/C^MCC^. To further test whether APC/C SUMOylation reduces the affinity of APC/C^Cdc20^ for the MCC, we performed size exclusion chromatography using either SUMOylated or non-SUMOylated APC/C mixed with the MCC and Cdc20 (Figures 6C–6E; Figure S7D). Non-SUMOylated APC/C^Cdc20^ robustly bound MCC as judged by a clear shift in its elution profile. In contrast, SUMOylated APC/C^Cdc20^ produced a broadened profile, indicative of a weaker interaction with the MCC. This difference is clearly seen in a plot of absorption difference between APC/C^Cdc20^ alone and APC/C^Cdc20^ incubated with the MCC (Figure S7E). We detected formation of the APC/C^Cdc20^-MCC complex in both samples, but with reduced MCC bound to SUMOylated APC/C^Cdc20^. Additionally, we analyzed the entire elution of two APC/C^Cdc20^ samples with MCC by blotting against BubR1 (Figures 6D and 6E). The SUMOylated APC/C^Cdc20^ sample had significantly more unbound BubR1 compared to non-SUMOylated APC/C^Cdc20^. Lastly, we analyzed peak fractions where the APC/C^Cdc20^-MCC complex was present, using immunoblotting against APC2, Cdc20, and all MCC components (Figure S7F, right). Whereas the levels of APC2 were the same in both samples, lower levels of BubR1, Bub3, and Mad2 were detected in the SUMOylated APC/C^Cdc20^ sample. The levels of Cdc20 appeared to be more similar in both samples, presumably because Cdc20 also binds as a coactivator (i.e., Cdc20^A^). Taken together, these data are consistent with the hypothesis that SUMOylation reduces the affinity of APC/C^Cdc20^ for the MCC.

### SUMOylation attenuates APC/C^Cdc20^ inhibition by the MCC

**Figure 7:**
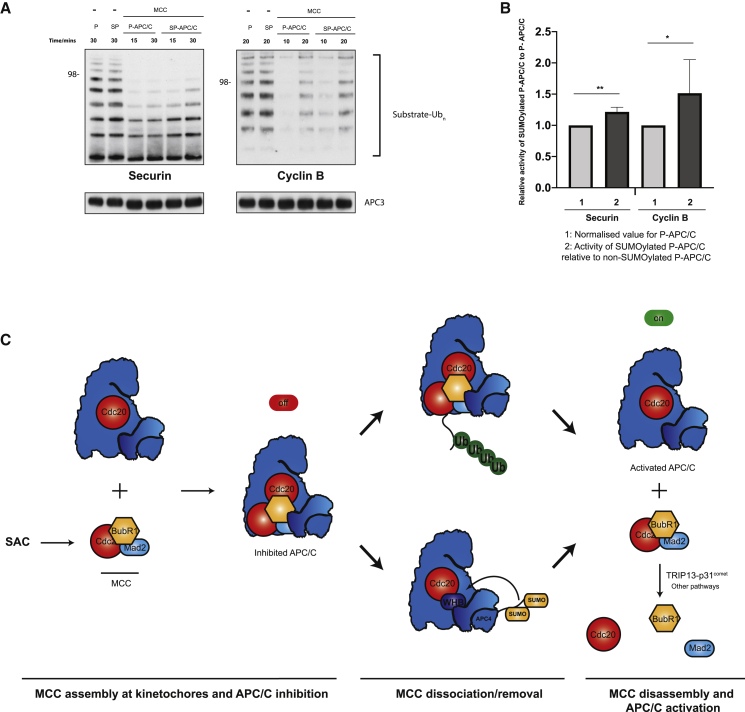
APC/C SUMOylation attenuates MCC inhibition of substrate ubiquitination by APC/C^Cdc20^ (A) Comparison of SUMOylated and non-SUMOylated APC/C ubiquitination activity toward securin and cyclin B using 150 nM UbcH10 supplemented with 300 nM Ube2S in the presence and absence of the MCC. Ubiquitination assay was performed as described in STAR Methods with the difference that MCC reactions were supplemented with 60 nM MCC and 30 nM Cdc20 was used. Samples with securin as a substrate were analyzed by immunoblotting against securin, whereas samples with cyclin B as the substrate were analyzed by immunoblotting against the His-tag of ubiquitin. (B) Quantification of (A) as described in Figure 5B. (C) Model of how APC/C becomes rapidly activated when the SAC is silenced. In a well-established mechanism, the MCC is auto-ubiquitinated, reducing its affinity for the APC/C, promoting its dissociating from the APC/C. In the SUMOylation-dependent mechanism, SUMO-2 displaces WHB domain from APC2–APC4 interface and further stabilizes it in the new position contacting APC10. This repositioned APC2^WHB^ directly blocks the MCC binding site on the APC/C. The dissociated MCC is then disassembled by TRIP13-p31^comet^, resulting in complete and rapid APC/C reactivation, triggering anaphase onset.

Given our observations that SUMOylation reduced the affinity of APC/C^Cdc20^ for the MCC, we tested whether SUMOylation would promote enhanced substrate ubiquitination in the presence of the MCC. We used securin and cyclin B as substrates because their ubiquitination is inhibited by the MCC (Zhang et al., 2019). Although, as mentioned earlier, there was no difference in the ubiquitination of both substrates in the absence of the MCC with UbcH10 and Ube2S (Figures 5E and 5F), in the presence of the MCC, SUMOylated APC/C^Cdc20^ ubiquitinated both substrates more efficiently than did non-SUMOylated APC/C^Cdc20^ (Figures 7A and 7B). This is consistent with our observations that SUMOylation reduced the affinity of APC/C^Cdc20^ for the MCC. However, the activity of SUMOylated APC/C^Cdc20^ was still suppressed by the MCC (Figure 7A). Thus, SUMOylation provided only partial relief of MCC suppression. In this regard, SUMOylation appears to function more similarly to MCC released by auto-ubiquitination, which also reduces the affinity of the MCC for APC/C^Cdc20^. Interestingly, MCC auto-ubiquitination does not allow anaphase substrate ubiquitination in the presence of the MCC (Miniowitz-Shemtov et al., 2010), whereas our data show that APC/C^Cdc20^ SUMOylation at least partially alleviates MCC inhibition.

## Discussion

This study allows us to better understand the mechanism of SAC inactivation in human cells (Figure 7C). APC/C^Cdc20^ is suppressed by the MCC during prophase, prometaphase, and metaphase. During these periods, the MCC is constantly generated at improperly attached kinetochores. Simultaneously, the MCC is constantly removed from the APC/C by two pathways. In the first well-established pathway, the APC/C^Cdc20^ auto-ubiquitinates the MCC, which reduces its affinity for the APC/C^Cdc20^ (Reddy et al., 2007; Uzunova et al., 2012). In the second pathway described in this study, SUMOylation promotes structural rearrangements of APC/C^Cdc20^ that also reduces its affinity for the MCC. Both of these pathways generate a pool of free MCC that can be disassembled by the TRIP13-p31^comet^ pathway (Eytan et al., 2014; Habu et al., 2002; Teichner et al., 2011; Xia et al., 2004). Once all kinetochores have attached to mitotic spindles, the MCC is no longer generated and the pool of free MCC is completely disassembled. Unlike the auto-ubiquitination activity of APC/C^Cdc20^ toward MCC, however, SUMOylation can be temporally and spatially regulated, for example by APC4 phosphorylation, allowing fine-tuning of APC/C activity in cells throughout mitosis. Our work also shows how SUMOylation of large multi-subunit protein complexes induces large conformational changes that regulate protein function.

Our results are consistent with previous studies of APC/C SUMOylation (Lee et al., 2018), where APC/C SUMOylation was suggested to peak at the metaphase-to-anaphase transition. How this is regulated is unknown. Our data explain the delay of anaphase onset observed in SUMOylation-defective APC/C mutant cells (Eifler et al., 2018; Lee et al., 2018), due to an impaired ability to reactivate APC/C^Cdc20^ rapidly once the SAC was silenced, and thus our results are consistent with the delay in degradation of cyclin B in SUMOylation-deficient APC/C mutant cells (Lee et al., 2018). Furthermore, our findings explain why SUMOylation-defective APC/C does not exhibit an *in vivo* phenotype in the presence of reversine when no MCC is formed, and why SUMOylation plays no additional roles apart from APC/C^Cdc20^ reactivation. Lastly, given that the repositioned WHB domain is in close proximity to the D-box recognition site on APC10, we cannot exclude the possibility that this would have an effect on at least some APC/C substrates.

We show that SUMO interacts with the APC/C in at least two ways: through a SIM on APC2, and by binding to the APC2–APC4 interface. The SIM on APC2 is important for APC/C SUMOylation but dispensable for APC2^WHB^ repositioning to contact APC10. Thus, the SIM on APC2 could provide a docking site for SUMO-loaded Ubc9 to enable preferential and efficient SUMOylation of the APC4 C terminus. Our findings that the APC2 SIM is required for optimal APC/C SUMOylation agree with Lee et al. (2018), who found that mutating APC2 SIM delayed the metaphase-to-anaphase transition, although less severely than a SUMOylation-deficient APC/C.

A similar EM density to our APC2^WHB^ density positioned below APC10 was observed previously in a cryo-EM structure of the APC/C in complex with Ube2S (Brown et al., 2014). Ube2S interacts with the APC/C at the APC2–APC4 interface through a C-terminal LRRL motif (Brown et al., 2016; Chang et al., 2015). Thus, a likely explanation for the density below APC10 in the Brown et al. (2016) study is that association of the Ube2S LRRL motif to its binding site at the APC2–APC4 interface displaced APC2^WHB^, promoting its relocation to APC10. Interestingly, the additional density at the APC2–APC4 interface that we assign to SUMO-2 slightly overlaps the LRRL motif binding site, suggesting that binding at this interface might be at least partially responsible for repositioning APC2^WHB^. It is unclear, however, how this would occur mechanistically. Two mechanisms to explain APC2^WHB^ repositioning are possible. In the first mechanism, which we term the multi-site binding model, APC2^WHB^ has weak affinity for both the APC2–APC4 site and APC10, and either the LRRL motif or SUMO-2 binding outcompetes APC2^WHB^ from the APC2–APC4 interface, forcing it to adopt a new position below APC10. The evidence for this model is the lack of a clear structural difference in APC10 with or without APC2^WHB^ binding. However, our EM maps of SUMOylated APC/C also contain an additional density below APC2^WHB^ when it is positioned below APC10 (Figure 3D), suggesting an alternative mechanism in which APC2^WHB^ is mobile and SUMOylation of APC/C directly stabilizes APC2^WHB^ in its new position below APC10 (mobile model). These two models are not mutually exclusive, and it is possible that both mechanisms contribute to stabilization of APC2^WHB^ in its position below APC10, as evidenced by the positioning of APC2^WHB^ below APC10 in the APC/C-Ube2S complex (Brown et al., 2016). The observation that Ube2S, an elongating E2 enzyme, could also reposition APC2^WHB^, which is essential for the function of the initiating E2 UbcH10 (Ube2C), might provide an elegant mechanism for switching the preference between these two E2s.

APC2^WHB^ emerges as a crucial and mobile element of the APC/C. Existing structures show that APC2^WHB^ is able to occupy four distinct sites: (1) its normal catalytic position when it interacts with the backside of UbcH10 (Brown et al., 2015); (2) interaction with BubR1 of the MCC (Alfieri et al., 2016; Yamaguchi et al., 2016); (3) interaction with APC10 described here; and (4) positioning at the APC2–APC4 interface (Alfieri et al., 2020). In all of these instances, APC2^WHB^ plays a role in regulating APC/C activity during the SAC.

What remains unclear is how the relatively low levels of SUMOylated APC/C that we and others detect in cells results in a delayed anaphase onset (Eifler et al., 2018; Lee et al., 2018). One explanation could be that the actual levels of SUMOylated APC/C in cells are higher than observed. A reason we might detect lower levels of SUMOylated APC/C during our assays is that most anti-APC4 antibodies tend to recognize the C-terminal region of the protein, the site of APC4 SUMOylation. We noticed that SUMOylated APC4 is detected less efficiently than the non-SUMOylated protein. All of these factors hamper estimation of the true levels of APC/C SUMOylation *in vivo*. Another explanation is that low levels of SUMOylation might be sufficient to remove MCC *in vivo* at the correct sub-cellular locations to robustly reactivate APC/C and initiate anaphase onset. Consistent with this hypothesis is the observation that the level of Cdc20 ubiquitination *in vivo* is also relatively low despite it being an important mechanism for MCC release (Mansfeld et al., 2011). Lastly, an important concept in SUMO signal transduction is target group modification (Psakhye and Jentsch, 2012). According to this concept, the effects of SUMO modification on individual target proteins are frequently modest or cannot even be detected. Nevertheless, by co-modifying larger sets of target proteins, the overall consequence of SUMO modification on cellular processes is highly significant. A modest biological effect of SUMO modification on APC4 is consistent with this concept. Nevertheless, the overall effect of SUMO modification on mitosis and cell cycle progression is drastic, due to the large set of target proteins that are co-regulated (Eifler and Vertegaal, 2015).

## STAR★Methods

### Key resources table

**Table:** 

REAGENT or RESOURCE	SOURCE	IDENTIFIER
**Antibodies**		

β-Actin (C4) HRP conjugated	Santa Cruz Biotechnology	sc-47778
Mouse monoclonal anti 6xHis	Clontech	631212
Rabbit polyclonal to APC4	Abcam	Ab72149
Rabbit polyclonal to APC4 for de-SUMOylation assays	Bethyl	A301-176A
Rat monoclonal to tubulin (YL1/2)	Abcam	Ab6160
Rabbit monoclonal to securin (19H16L48)	ThermoFisher	700791
Rabbit polyclonal to securin	Cell Signaling Technology	D2B60
Rabbit polyclonal anti-phospho Histone H3 (Ser10), Mitosis Marker	Merck	06-570
Mouse Anti-Bub3	BD Biosciences	611730
Mouse Anti-MAD2	BD Biosciences	610679
Rabbit polyclonal to SUMO2/3	Abcam	Ab3742
Mouse monoclonal to SUMO2/3 for de-SUMOylation assays and U2OS experiments	University of Iowa	AB_2198421
Rabbit monoclonal to Cdc23 (APC8)	Abcam	Ab182003
Mouse monoclonal to BubR1 (8G1)	Santa Cruz Biotechnology	sc-47744
Rabbit polyclonal to p55 CDC (Cdc20) (h-175)	Santa Cruz Biotechnology	sc-8358
Rabbit monoclonal to Cyclin A2	Cell Signaling Technology	E1D9T
Goat polyclonal to Ube2I (Ubc9)	Abcam	Ab21193
Rabbit polyclonal to RanBP2	Abcam	Ab112061
Rabbit monoclonal to APC3	Cell Signaling Technology	12530s
Rabbit polyclonal to APC2	Cell Signaling Technology	12301s
HRP conjugated sheep anti-mouse	GE Healthcare	NXA931V
HRP conjugated donkey anti-rabbit	ThermoFisher	SA1-200
HRP conjugated donkey anti-goat	Promega	V8051
HRP conjugated anti-rat	Santa Cruz Biotechnology	Sc-2032

**Chemicals, peptides, and recombinant proteins**		

Biotin	Sigma-Aldrich	B4501
Desthiobiotin	Sigma-Aldrich	D1411
Thymidine	Sigma-Aldrich	T9250
S-trityl-L-cysteine (STLC)	Sigma-Aldrich	164739
CDK1/2 inhibitor iii	Enzo Life Sciences	ALX-270-442-M001
Complete™ EDTA-free protease inhibitors	Roche	11873580001
Phenylmethanesulfonyl fluoride (PMSF)	Sigma-Aldrich	78830

**Deposited data**		

CryoEM map for SUMOylated APC/C with repositioned WHB domain	This study	EMD-10536
CryoEM map for SUMOylated APC/C with repositioned WHB domain, body 2 after multi body refinement	This study	EMD-10536
CryoEM map for SUMOylated APC/C with additional density at APC2-APC4 interface	This study	EMD-10538
Model of SUMOylated APC/C with repositioned WHB domain	This study	PDB ID: 6TNT

**Experimental models: Cell lines**		

HEK293 FlpIn-TRex	Invitrogen	R78007
FreeStyle 293-F Cells	Invitrogen	R79007
U2OS cells	Hendriks and Vertegaal., 2016b	N/A
U2OS cells with HIS10-SUMO2 stably intergrated	Hendriks and Vertegaal., 2016b	N/A
Sf9 cells	Invitrogen	11496015
High Five cells	Invitrogen	B85502

**Oligonucleotides**		

SiRNA for LuciferaseGL2 (AACGTACGCGGAATACTTCGA)	Sigma-Aldrich	This study
siRNA for Ubc9a (CAAAAAATCCCGATGGCAC)	Sigma-Aldrich	This study
siRNA for Ubc9b (TTCTTGCCAAACCAATCCCTT)	Sigma-Aldrich	This study
siRNA for RanBP2 (5677 start site)	Sigma-Aldrich	SASI_Hs01_00221992

**Recombinant DNA**		

pGEX-6P-1	EMBL	N/A
pETM11	EMBL	N/A
pFastBac-6xHis-Kif18B	This study	N/A
APC/C with APC2^ΔWHB^ (APC2^N1-L765)^	This study	N/A
SIM APC/C mutant (V728A, L729A and I730A OF APC2)	This study	N/A
SUMO2ΔNTD (Δ 1-15, sequence from D16-G93)	N/A	This study
SUMO1ΔNTD (Δ 1-19, sequence from E20-G97)	N/A	This study

**Software and algorithms**		

GraphPad Prism	GraphPad Software	https://www.graphpad.com/
RELION 3.0	MRC-LMB	https://www3.mrc-lmb.cam.ac.uk/relion/index.php/Main_Page
UCSF Chimera	UCSF	https://www.cgl.ucsf.edu/chimera/ (Yang et al., 2012)
UCSF Chimera X	UCSF	https://www.cgl.ucsf.edu/chimerax/ (Goddard et al., 2018)
Pymol	Schrödinger	https://pymol.org/2/
Coot	MRC-LMB	https://www2.mrc-lmb.cam.ac.uk/personal/pemsley/coot/
Jalview	Jalview	http://www.jalview.org (Waterhouse et al., 2009)
Excel	Microsoft	https://www.microsoft.com/en-gb/microsoft-365/excel
ImageJ	NIH	https://imagej.nih.gov/ij/index.html
Adobe Illustrator	Adobe	https://www.adobe.com/uk/creativecloud.html

**Other**		

Insect Express	Lonza	B12-730Q
Sf900-II SFM	Life Technologies	10902096
DMEM high glucose	Thermo Fisher	11965092
Tetracycline-free Fetal Bovine Serum (FBS)	PAN Biotech	P30-3601
GlutaMAX Supplement	ThermoFisher	35050038
FreeStyle 293 Expression Medium	ThermoFisher	12338018
Lipofectamine RNAiMAX Transfection Reagent	ThermoFisher	13778075
Opti-MEM I Reduced Serum Medium	ThermoFisher	31985062
Dynabeads M-280 Streptavidin	ThermoFisher	11205D

### Resource availability

#### Lead contact

Further information and requests for resources and reagents should be directed to and will be fulfilled by the lead contact, David Barford (dbarford@mrc-lmb.cam.ac.uk).

#### Materials availability

All unique/stable reagents generated in this study are available from the lead contact with a completed Materials Transfer Agreement.

#### Data and code availability

The cryoEM maps have been deposited to the Electron Microscopy Data Bank (EMDB) with accession numbers EMDB: EMD-10536, EMD-10538. The protein model has been deposited to the Protein Data Bank (PDB) with accession number PDB: 6TNT.

### Experimental model and subject details

XL-1 Blue Cells (Agilent) were used to propagate recombinant DNA vectors. BL21 (DE3) Star Cells (Thermo Fisher) were used for all bacterial protein expression. High five insect cells (Invitrogen) were used for all insect cell protein expression. HEK293 FlpIn-TRex cells (Invitrogen) cells were used to detect APC4 SUMOylation *in vivo*. U2OS cells with HIS10-SUMO2 stably integrated were used as described in Hendriks and Vertegaal., 2016b.

### Method details

#### Expression and purification of recombinant human APC/C, MCC, and coactivators

Human APC/C was expressed using the baculovirus/insect cell system and purified as described (Zhang et al., 2013). In brief, the APC4 C terminus was fused with a TEV-cleavable StrepIIx2 tag and the entire complex was expressed in Hi5 cells for 48-72 h. Cells were re-suspended in APC/C wash buffer (50 mM Tris.HCl pH 8.0, 250 mM NaCl, 2 mM DTT and 5% glycerol) supplemented with 2 mM benzamidine, 1 mM EDTA, 0.1 mM PMSF, Complete EDTA-free protease inhibitor tablets (Roche) and benzonase. The APC/C was subsequently further purified using a Resource Q anion exchange column and by gel filtration into APC/C gel filtration buffer (20 mM HEPES pH 8.0, 150 mM NaCl and 0.2 mM TCEP). The MCC was purified as described (Alfieri et al., 2016). In brief, StrepIIx2 tagged BubR1 was co-expressed with Cdc20-MBP fusion, Mad2 and Bub3 and purified as described for APC/C above. Full length Cdh1 and Cdc20 were purified as described (Chang et al., 2015; Zhang et al., 2016).

#### Expression and purification of SUMOylation and ubiquitination pathway proteins

SUMO constructs (SUMO-1, SUMO-2, SUMO-2ΔNTD, SUMO-1ΔNTD) were cloned into pETM11 as a fusion with TEV cleavable N-terminal 6xHis tag. Proteins were expressed in *E. coli* BL21 star cells at 18°C overnight. Proteins were purified in lysis buffer containing 50 mM Tris.HCl pH 7.5, 500 mM NaCl, 5% glycerol and 10 mM imidazole supplemented with PMSF and EDTA-free protease inhibitor cocktail using HiTRAP TALON (GE Healthcare) columns. The 6xHis tag was either cleaved overnight using TEV or retained for some SUMO-1 and SUMO-2 samples. Uncleaved 6xHis-SUMO-1 and 6xHis-SUMO-2 were further purified using gel filtration in APC/C gel filtration buffer (20 mM HEPES pH 8.0, 150 mM NaCl, 0.5 mM). Cleaved proteins were passed through a HiTRAP TALON (GE Healthcare) column again where the flow through was collected and proteins further purified using gel filtration in APC/C gel filtration buffer.

Uba2-Aos1, Ubc9 and UbcH5 (Ube2D3) were cloned into the pGEX6p1 vector as N-terminal GST-tag fusions. Both proteins were expressed in *E. coli* BL21 star cells at 18°C overnight. Cell pellets were resuspended in lysis buffer containing 50 mM Tris pH 7.5, 150 mM NaCl, 5% glycerol and supplemented with PMSF and Complete EDTA-free protease inhibitor cocktail (Roche) and purified using Glutathione Sepharose 4b (VWR). Proteins were eluted by GST-tag cleavage and further purified using gel filtration in APC/C gel filtration buffer.

#### Expression and purification of APC/C substrates

Cyclin B-Cdk-Cks complex was purified as described (Zhang et al., 2019). Securin was purified as described (Alfieri et al., 2016). Hsl1 peptide was purified as described (Chang et al., 2015). Human Kif18B (10-828) was subcloned into the pFastBac HTa vector with an N-terminal 6xHis-tag. Kif18B was expressed in Hi5 insect cells. The cells were harvested after 48 h and stored in −80°C until purification. Protein was purified using HisTrap HP 5 mL columns (GE healthcare). The cells were lysed in 50 mM Tris pH 7.5, 300 mM NaCl, 5% glycerol, 1 mM TCEP, 30 mM imidazole (wash buffer) supplemented with PMSF and Complete EDTA-free protease inhibitor cocktail tablets (Roche). Protein was eluted in 300 mM imidazole pH 7.5. Sample was then diluted by half with 20 mM HEPES pH 7.5, 5% glycerol and 1 mM TCEP and no salt and loaded onto a Resource S column. The sample was washed with 5 column volumes of the Resource S column with 2 mM ATP, 5 mM MgCl_2_ and 20 mM KCl. Protein was eluted with a gradient of NaCl up to 500 mM, concentrated and further purified by gel filtration on a Superdex 200 column in 20 mM HEPES pH 7.5, 150 mM NaCl and 1 mM TCEP.

#### *In vivo* cell cycle arrest experiments

Flp-In T-REx HEK293 cells were grown in DMEM media supplemented with FBS and Pen/Strep antibiotics. Cells were arrested using 1 μM thymidine, released into fresh media the next day and then arrested again using 1 μM thymidine to give early S-phase arrested cells. For mitotically arrested cells, double thymidine arrested cells were released for 4-5 h into fresh media, and then arrested using 5 μM STLC overnight. APC4 was visualized with polyclonal anti-APC4 rabbit antibody (Abcam ab72149).

Cells were lysed in APC/C lysis buffer (50 mM HEPES pH 8, 200 mM NaCl, 5 mM EGTA, 0.1% Triton X-100, 1 mM NEM, Complete EDTA-free protease inhibitor cocktail (Roche), 1 mM PMSF and benzonase) on ice for 30 min after which sample loading buffer was added. Cell lysates were run on 4%–12% NuPAGE Bis-Tris protein gels and blotted onto nitrocellulose membrane.

#### *In vivo* RNAi experiments

Flp-In HEK293 T-REx cells were grown in DMEM media supplemented with FBS and Pen/Strep antibiotics. Cells were plated in 24-well plates and transfected with Lipofectamine RNAiMAX reagent (Thermo Fisher Scientific) at confluency of 0.8. 20 nM siRNAs were used for Ubc9 depletion and 40 nM siRNA was used for RanBP2 depletion. Cells were left for 48 h, after which STLC was added and cells were incubated for additional 16 h to enrich for mitotically arrested cells. For RanBP2 detection, cells were split and one part was lysed using RIPA buffer supplemented with 1 mM NEM, Complete EDTA-free protease inhibitor cocktail (Roche), 1 mM PMSF and benzonase. The cell lysate samples for RanBP2 blot were run on 3%–8% Tris-Acetate protein gels and samples were blotted onto PVDF membrane.

#### Purification of HIS10-SUMO2 from U2OS cells

Purification of His10-SUMO2 was performed as described (Hendriks and Vertegaal, 2016b). Briefly, U2OS cells stably expressing His10-SUMO2 were lysed in 10 pellet volumes of 6 M guanidine-HCL, 100 mM sodium phosphate, 10 mM Tris at pH 8.0. Sonication of the lysates was performed 2 times for 10 s and subsequently, 5 mM β-mercaptoethanol and 50 mM imidazole pH 8.0 were added. Ni-NTA beads (QIAGEN, 30210) were washed and subsequently added to the lysates and samples were kept rotating overnight at 4°C. Ni-NTA beads were washed with wash buffers as described (Hendriks and Vertegaal, 2016b). Purified proteins were eluted in elution buffer (7 M urea, 100 mM sodium phosphate, 10 mM Tris and 500 mM imidazole pH 7.0).

#### Immunoblotting of HIS10-SUMO2 from U2OS cells

Total cell lysates were prepared using SNTBS buffer (2% SDS, 1% NP40, 50 mM Tris pH 7.5, 150 mM NaCl) and boiled at 100°C for 10 min. Samples were loaded onto 4%–12% gradient gels Bolt Bis–Tris (Thermo Fisher Scientific). Protein transfer was performed using Amersham Protran Premium nitrocellulose membranes (Sigma Aldrich). Blocking of membranes was performed with 5% milk in PBS-T (0.05% Tween) for 60 min and subsequently, primary antibodies were added and blots were incubated overnight at 4°C. Donkey anti-rabbit IgG-HRP and goat anti-mouse IgG-HRP secondary antibodies were diluted 1:2500 in PBS-T (0.05% Tween) containing 5% milk and detection was performed by chemiluminescence using Pierce ECL Plus Western Blotting substrate (32132, Thermo Fisher Scientific).

#### FACS analysis

Cells were harvested by trypsinization (a-synchronous cells) or mitotic shake-off (STLC treated cells) and fixed with ice-cold 70% ethanol overnight at −20°C. Cells were washed twice with PBS-T (0.05% Tween), and subsequently twice with PBS-T containing 1% BSA. Cells were incubated with antibody against MPM2 (mouse, Sigma-Aldrich) for 4 h at 4°C, including re-suspension after 2 h in PBS-T containing 1% BSA. Washing steps were repeated. Cells were incubated with secondary anti-mouse antibody coupled to FITC (DAKO, F0479) in PBS-T containing 1% BSA for 1 h at room temperature. Washing steps were repeated. Cells were re-suspended in 100 μL buffer containing propidium iodide and RNase overnight at 4°C. The gating strategies for flowcytometry are provided in the supplementary information.

#### SUMOylation assay

The SUMOylation assay was performed with 0.1 μM APC/C, 0.18 μM Uba2-Aos1, 1.6 μM Ubc9 (or 0.8 μM for RanBP2 assay), 18 μM SUMO-2 or SUMO-1 in 20 mM HEPES pH 8, 50 mM NaCl, 0.5 mM TCEP and 5 mM MgCl_2_. Reaction mixtures were incubated at room temperature for various time points indicated in figures and terminated by adding SDS/PAGE loading buffer. Reactions were analyzed by 4%–12% NuPAGE Bis-Tris gels followed by western blotting onto nitrocellulose membrane and immunoblotting with an antibody against APC4 (Abcam ab72149).

#### *In vitro* de-SUMOylation assay

De-SUMOylation was performed with 0.5 μM APC/C or 0.5 μM SUMOylated APC/C and 0.5 μM SENP2-catalytic domain in a buffer containing 25 mM Tris pH 8, 150 mM NaCl, 0.1% Tween-20 and 2 mM DTT in a total reaction volume of 20 μL. Reaction mixtures were incubated for 2 h at 37°C and as a control (SUMOylated) APC/C in reaction buffer without SENP2 at 4°C. The reaction was terminated by adding sample buffer. Samples were size-separated on Novex Bolt 4%–12% Bis-Tris Plus. Proteins were transferred to Amersham Protran Premium nitrocellulose membranes. Membranes were blocked in PBS-T (0.05% Tween) containing 5% milk for 60 min. Primary antibodies were diluted in PBS-T (0.05% Tween) and incubated with the membranes at 4°C overnight. APC4, SUMO2/3, Donkey anti-rabbit IgG-HRP and goat anti-mouse IgG-HRP secondary antibodies were diluted 1:2500 in PBS-T (0.05% Tween) containing 5 % milk and detected using chemo luminescence with Pierce ECL Plus western blotting substrate (32132, Thermo Fisher Scientific).

#### Ubiquitination assays

Ubiquitination assays were performed with 60 nM recombinant human APC/C, 90 nM UBA1, 150 nM UbcH10 or 300 nM UbcH5, 300 nM Ube2S, 70 μM ubiquitin, 2 μM substrate, 5 mM ATP, 0.25 mg ml^−1^ BSA, and different concentrations of purified human Cdc20 or Cdh1 in a 10 μL reaction volume with 40 mM HEPES pH 8.0, 10 mM MgCl_2_ and 0.6mM DTT (figure legends indicate the exact coactivator concentration used in each assay). 15 μM CDK1/2 III inhibitor was also added to reactions that contained cyclin B. Reaction mixtures were incubated at room temperature for various time points and terminated by adding SDS/PAGE loading dye. Reactions were analyzed by 4%–12% NuPAGE Bis-Tris gels followed by western blotting with an antibody against the His-tag of ubiquitin.

To test the activity of APC/C and SUMOylated APC/C toward the Cdc20^MCC^ from individually purified wild-type MCC ubiquitination reactions were performed with 200 nM of recombinant human APC/C, 200 nM of recombinant human Cdc20 and 200 nM of recombinant human MCC in the presence of 300 nM of either UbcH5 or UbcH10. To test the activity of APC/C toward cyclin B and securin in the presence of MCC, 60 nM recombinant human APC/C was used with 60 nM of MCC and 30 nM of Cdc20 and either 2 μM of securin or 1 μM of cyclin B.

#### Size exclusion chromatography

For binding studies between APC/C, APC/C^ΔSIM^ and SUMO-2, APC/C or APC/C^ΔSIM^ at 1 μM were incubated with 60 μM of SUMO-2 on ice for 30 min and then samples were injected onto a Superose 6 Increase 3.2/300 gel filtration column. For binding studies between phosphorylated APC/C, SUMOylated and phosphorylated APC/C and MCC with Cdc20, MCC and Cdc20 were added in 1.5 molar excess to APC/C, incubated for half an hour on ice and then injected onto a Superose 6 Increase 3.2/300 gel filtration column. 50 μL fractions were collected in both cases.

#### Electron microscopy

For cryo-EM, 2.5 μL aliquots of the sample at ∼0.15 mg ml^−1^ were applied to Quantifoil 3.5/1 grids coated with a layer of continuous carbon film (approximately 50 Å thick). Grids had been treated with a 9:1 argon:oxygen in a plasma cleaner for 20 to 40 s before use. Following application of sample, the grids were incubated for 30 s at 4 °C and 100% humidity before blotting for 5 s and plunging into liquid ethane using an FEI Vitrobot III. Cryo-EM micrographs were collected with an FEI Titan Krios electron microscope at an acceleration voltage of 300 kV and Falcon III direct detector in electron counting mode. Micrographs were taken using EPU software (FEI) at a nominal magnification of 81000, yielding a pixel size of approximately 1.1 Å per pixel at specimen level. A total exposure time of 59.98 s was used at a dose rate of 0.5-0.6 electrons per pixel. Defocus range was set at −2.0 to −4.0 μm.

#### Image processing

Image processing was performed with RELION 3.0 (Zivanov et al., 2018). The initial steps including motion correction, CTF estimation, particle picking and particles sorting by *Z*-score and 2D classification were performed as described (Chang et al., 2015). Selected particles were used for a first round of 3D classification with a global search and a sampling angular interval of 7.5°, using a 60 Å low-pass filtered apoAPC/C EM map as a reference. Poorly characterized 3D classes, with poorly recognizable features, were discarded at this stage and the remaining particles were refined and corrected for beam-induced particle motion using particle polishing in RELION. Polished particles were used for another round of 3D classification where the best particles were selected. The reconstruction generated from all the polished particles, low-pass filtered at 40 Å, was used as reference.

Focused 3D classification was used to improve densities near the APC10 and APC4 subunits. For improvement of density at the APC2–APC4 interface, a large mask including most of APC4 and part of APC2 was generated. This mask was used to subtract APC/C signal and classify the APC2–4 region without image alignment. This allowed identification and isolation of the partially activated apoAPC/C. The remaining classes were combined and classified using a tight mask that included the APC4 WD40 domain and cullin domain of APC2 only. This procedure was repeated twice to improve the classification. This allowed isolation of classes with high SUMO-2 occupancy. Selected classes were reverted to full particles and refined to give overall maps.

To increase the occupancy of density below APC10, a mask that included APC10, part of APC1 and part of APC3 was used for focused 3D classification without image alignment using subtracted APC/C. This procedure was repeated twice to improve the classification. Classes were selected where the density was clearly seen, reverted to full particles and refined. The APC/C then was divided into three bodies and multi body refinement implemented in RELION 3.0 was used to improve angular alignments around APC10 and APC2^WHB^ region.

The apoAPC/C dataset from Zhang et al. (2016) was processed in an identical way to test whether the same structural rearrangements occur without SUMOylation.

EMDA software was used to generate a difference map between apoAPC/C and SUMOylated APC/C (Warshamanage and Murshudov, 2020).

#### Map visualization and model building

Figures were generated using PyMOL (The PyMOL Molecular Graphics System, Version 2.0 Schrödinger, LLC.), Chimera (Yang et al., 2012) and ChimeraX (Goddard et al., 2018). Models were built in Coot (Emsley et al., 2010) using existing structures from PDB. For fitting the WHB domain PDB ID 4YII was used, for fitting SUMO-2, PDB ID 1WM3 was used, for MCC-APC/C^Cdc20^ PDB ID 5LCW was used.

#### Sequence alignment

Sequence alignment was performed using Jalview (Waterhouse et al., 2009).

### Quantification and statistical analysis

#### Quantification of ubiquitination assays

Quantification was performed using ImageJ. Briefly, each lane was manually selected and the background signal from the lane that did not contain APC/C was subtracted. For the UbcH10 experiments in Figure 5, the top of the ubiquitin-substrate signal was used and whole lanes were used for all other experiments. Quantification was done over two independent gels each containing three time points, giving six points overall. A third technical repeat was performed but data from this repeat were not used for quantification. The ratios used for plotting histograms in Figures 5, 6, and 7 were obtained from either unmodified APC/C or SUMOylated APC/C divided by the corresponding time-point activity value of unmodified APC/C, which effectively shows the activity of SUMOylated APC/C compared to unmodified APC/C. The data were plotted using Prism 8. The bar height is the mean value with one standard deviation shown.

Statistical analysis was performed with Prism 8 (Graphpad). A two-tailed Student’s t test was used to calculate the significance. One star indicates significance smaller than 0.05, two stars indicate significance smaller than 0.01.

#### Quantification of SUMOylation assays

Quantification was performed using ImageJ. For each sample at a given time point, signal from both modified and unmodified APC4 was measured. The ratio was calculated of signal from modified APC4 divided by the signal from unmodified APC4. The graphs and statistical analysis was performed using Prism 8 (GraphPad). Each assay was performed in triplicate and each time point represents three independent values for each sample as mean with standard deviation.

#### Quantification of the level of *in vivo* APC4 SUMOylation

Quantification of the *in vivo* APC4 SUMOylation in mitosis was performed by measuring the band intensity of SUMOylated APC4 in each experiment and comparing it to the intensity of SUMOylated APC4 in asynchronous cells (AS) using ImageJ. Data represent the mean with one standard deviation (n = 3). n represents the number of repeats for this particular experiment. Statistical analysis was performed using unpaired Student’s t test with four stars indicating significance below 0.001 using Prism 8 (Graphpad).

## Supplemental information

Document S1. Figures S1–S7 and Table S1

Document S2. Article plus supplemental information
