# Supplementary material for: Molecular mechanisms of APC/C release from spindle assembly checkpoint inhibition by APC/C SUMOylation
Source: Cell Rep. 2021 Mar 30;34(13):108929. doi: 10.1016/j.celrep.2021.108929 (PMC8028313; doi:10.1016/j.celrep.2021.108929)
Supplement: Document S1. Figures S1–S7 and Table S1 [file mmc1.pdf]

Cell Reports, Volume *34*

Supplemental information

Molecular mechanisms of APC/C release from spindle assembly checkpoint inhibition by APC/C SUMOylation

Stanislau Yatskevich, Jessie S. Kroonen, Claudio Alfieri, Thomas Tischer, Anna C. Howes, Linda Clijsters, Jing Yang, Ziguo Zhang, Kaige Yan, Alfred C.O. Vertegaal, and David Barford

Figure S1

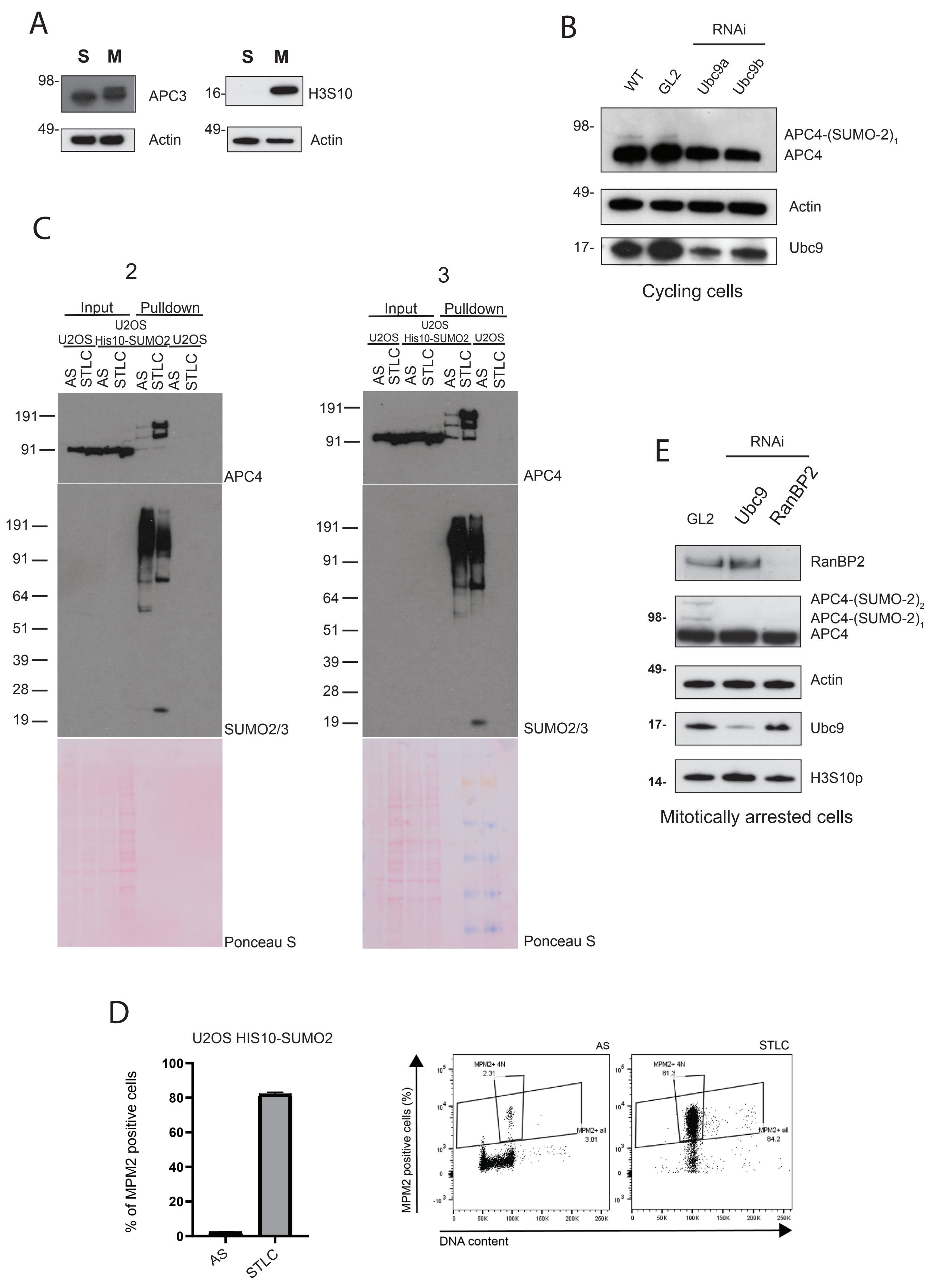
A
S M
98-
APC3
49-
Actin
S M
16-
H3S10
49-
Actin
B
RNAi
WT
GL2
Ubc9a
Ubc9b
98-
APC4-(SUMO-2)1
APC4
49-
Actin
17-
Ubc9
Cycling cells
C
2
Input
Pulldown
U2OS
U2OS
His10-SUMO2
U2OS
AS
STLC
AS
STLC
AS
STLC
AS
STLC
191
91
APC4
191
91
64
51
39
28
19
SUMO2/3
Ponceau S
3
Input
Pulldown
U2OS
U2OS
His10-SUMO2
U2OS
AS
STLC
AS
STLC
AS
STLC
AS
STLC
191
91
APC4
191
91
64
51
39
28
19
SUMO2/3
Ponceau S
E
RNAi
GL2
Ubc9
RanBP2
RanBP2
APC4-(SUMO-2)2
98-
APC4-(SUMO-2)1
APC4
49-
Actin
17-
Ubc9
14-
H3S10p
Mitotically arrested cells
D
U2OS HIS10-SUMO2
% of MPM2 positive cells
100
80
60
40
20
0
AS
STLC
AS
STLC
MPM2+ 4N
2.31
MPM2+ all
3.01
MPM2+ 4N
81.3
MPM2+ all
84.2
MPM2 positive cells (%)
DNA content

Figure S1. STLC arrested cells are efficiently arrested in mitosis and RanBP2 depletion abolishes APC/C SUMOylation bands *in vivo*. Related to figure 1.

(A) Cell cycle synchronization control for Figure 1A. Immunoblotting against APC3 (left panel) or phosphorylated Ser10 of histone H3 (right panel) shows that cells are efficiently arrested in mitosis with STLC. Lower panel: loading control.

(B) Cycling cells were analysed for APC4 SUMOylation. Ubc9 was depleted for 48 h using 20 nM siRNA after which the cells were harvested and analysed by immunoblotting against APC4. Loading was controlled by immunoblotting against actin and depletion was confirmed by immunoblotting against Ubc9.

(C) Repeats of experiments in Figure 1C. Method is described in Figure 1C and in Methods.

(D) Cell cycle synchronization of experiments in Figure 1C was verified by FACS analysis of DNA content and MPM2 (Mitotic Phosphoprotein Marker 2, anti-Cdk phosphorylated proteins) expression and shown in FACS plots (y-axis MPM2 positive cells, x-axis DNA content). Quantifications are shown in a bar graph. Data represent mean with one standard deviation (n=2).

(E) SUMOylated APC4 bands disappeared in cells treated with siRNAs against RanBP2 or Ubc9 but not in control cells, suggesting that APC/C SUMOylation depends on RanBP2. Proteins were depleted for 48h after which STLC was added and cells were left for additional 16 h before harvesting. 20 nM siRNA was used to deplete Ubc9 and 40 nM to deplete RanBP2. APC4 SUMOylation was detected by immunoblotting against APC4 and RNAi efficiency was assessed by immunoblotting against Ubc9 and RanBP2. Immunoblotting against phosphorylated histone H3 Ser10 (H3S10p) confirmed that the difference in APC4 SUMOylation is not due to cells being at different stages of the cell cycle.

Figure S2

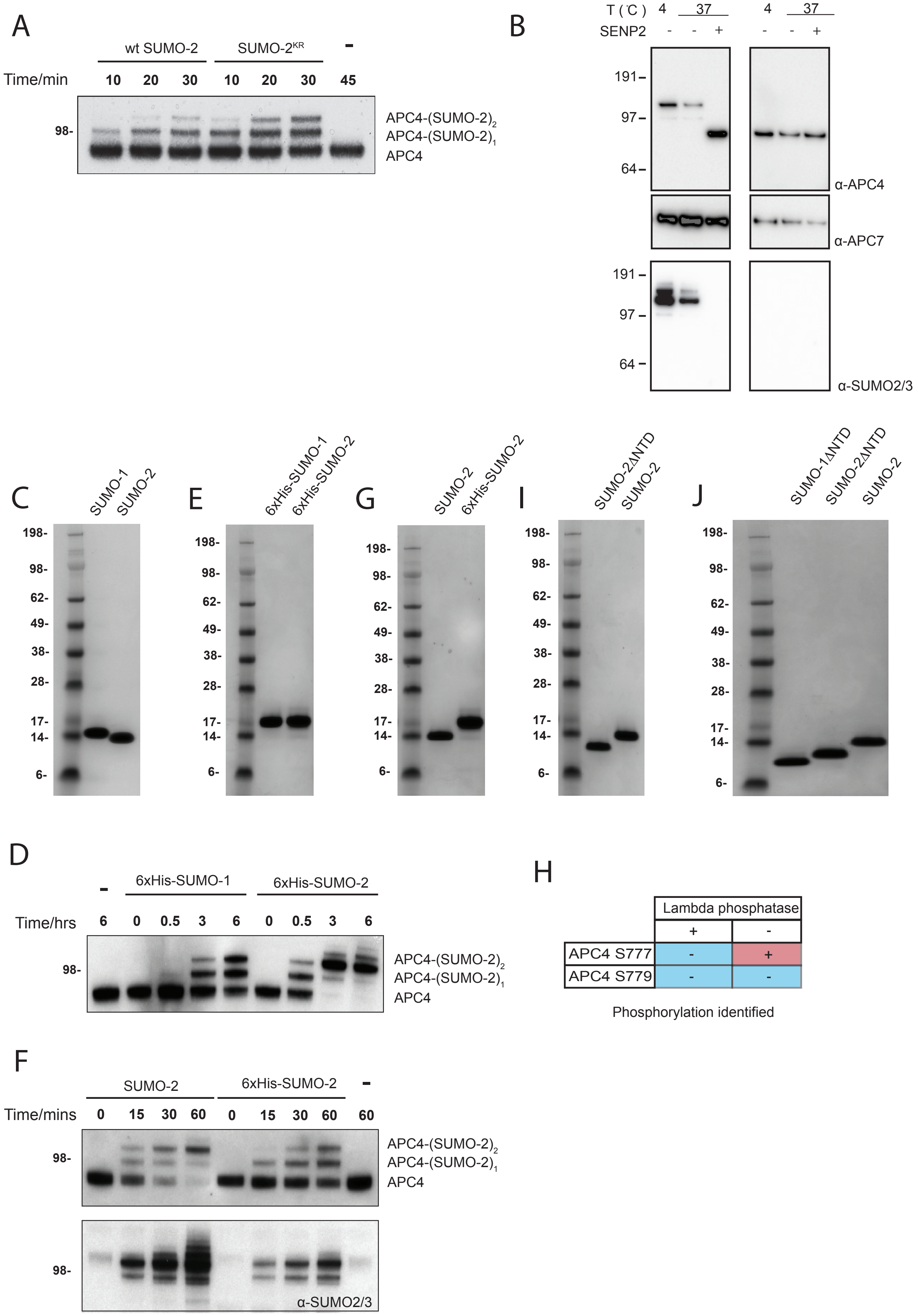

A
wt SUMO-2
SUMO-2KR
-
Time/min
10 20 30 10 20 30 45
98-
APC4-(SUMO-2)2
APC4-(SUMO-2)1
APC4
B
APC/C(SUMO2)2
apoAPC/C
T (˚C)
4 37 4 37
SENP2
- - + - - +
191-
97-
64-
α-APC4
α-APC7
α-SUMO2/3
C
SUMO-1
SUMO-2
E
6xHis-SUMO-1
6xHis-SUMO-2
G
SUMO-2
6xHis-SUMO-2
I
SUMO-2ΔNTD
SUMO-2
J
SUMO-1ΔNTD
SUMO-2ΔNTD
SUMO-2
198-
98-
62-
49-
38-
28-
17-
14-
6-
D
-
6xHis-SUMO-1
6xHis-SUMO-2
Time/hrs
6 0 0.5 3 6 0 0.5 3 6
98-
APC4-(SUMO-2)2
APC4-(SUMO-2)1
APC4
H
Lambda phosphatase
+ -
APC4 S777 - +
APC4 S779 - -
Phosphorylation identified
F
SUMO-2
6xHis-SUMO-2
-
Time/mins
0 15 30 60 0 15 30 60 60
98-
APC4-(SUMO-2)2
APC4-(SUMO-2)1
APC4
98-
α-SUMO2/3

Figure S2. Analysis of effects of the N-terminal 6xHis tag of SUMO-2 on the SUMOylation reaction and SUMO isoforms loading controls. Related to figure 2.

(A) A SUMOylation assay showed that APC/C SUMOylation by SUMO-2 and by a SUMO-2 mutant where all Lys were replaced with Arg (SUMO-2^{KR}) both produced two additional SUMOylation bands above APC4. The SUMOylation assay was performed as described in Methods and APC4 was detected by immunoblotting with antibodies against APC4.

(B) De-SUMOylation assay shows that SENP2 can remove SUMO from the APC/C. De-SUMOylation was performed with either 0.5 μM APC/C or 0.5 μM SUMOylated APC/C and 0.5 μM SENP2-catalytic domain as indicated, in a buffer containing 25 mM Tris (pH 8), 150 mM NaCl, 0.1% Tween-20 and 2mM DTT in a total reaction volume of 20 μL. APC4 and SUMO-2/3 were detected by immunoblotting with antibodies against APC4 and SUMO-2/3, respectively.

(C) SDS-PAGE gel of SUMO-1 and SUMO-2 loading controls used for Figure 2A stained with Coomassie Blue.

(D) APC/C SUMOylation by the 6xHis-SUMO-2 isoform is much faster than by 6xHis-SUMO-1 isoform. The SUMOylation assay was performed as described in Methods and APC4 was detected by immunoblotting with antibodies against APC4.

(E) SDS-PAGE gel of 6xHis-SUMO-1 and 6xHis-SUMO-2 loading controls used for Figure S2D stained with Coomassie Blue.

(F) APC/C SUMOylation by unmodified SUMO-2 isoform is much faster than by 6xHis-SUMO-2. The SUMOylation assay was performed as described in Methods and APC4 and SUMO-2/3 were detected by immunoblotting with antibodies against APC4 and SUMO-2/3, respectively.

(G) SDS-PAGE gel of SUMO-2 and 6xHis-SUMO-2 loading controls used for Figure S2F stained with Coomassie Blue.

(H) APC4 phosphorylation state summary at S777 and S779 before and after lambda phosphatase treatment as determined by mass spectrometry peptide analysis.

(I) SDS-PAGE gel of SUMO-$2^{\Delta NTD}$ and SUMO-2 loading controls used for Figure 2D stained with Coomassie Blue.

(J) SDS-PAGE gel of SUMO-$1^{\Delta NTD}$, SUMO-$2^{\Delta NTD}$ and SUMO-2 loading controls used for Figure 2E stained with Coomassie Blue.

Figure S3

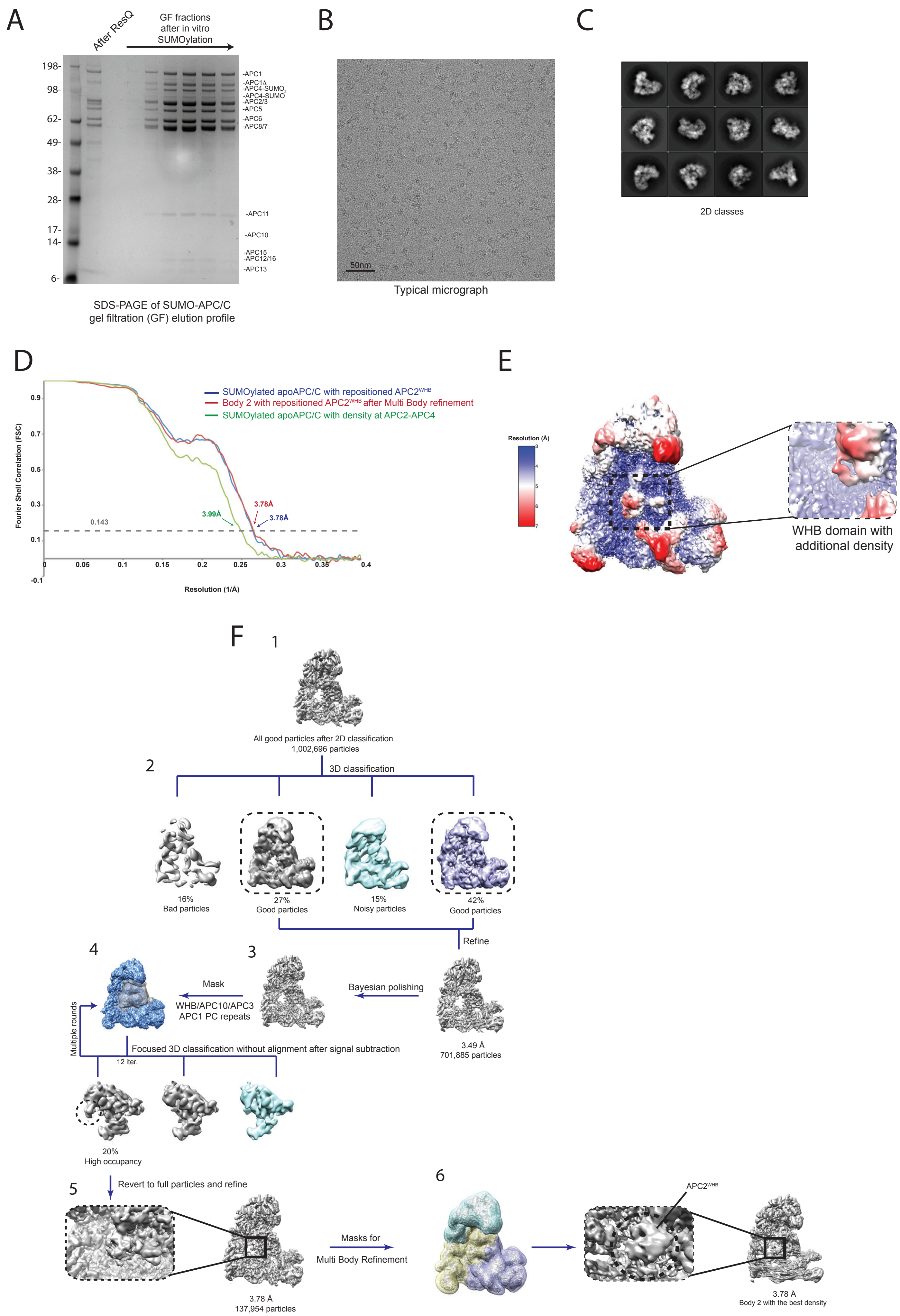
A
After ResQ
GF fractions after in vitro SUMOylation
198-
98-
62-
49-
38-
28-
17-
14-
6-
-APC1
-APC1A
-APC4-SUMO2
-APC4-SUMO
-APC2/3
-APC5
-APC6
-APC8/7
-APC11
-APC10
-APC15
-APC12/16
-APC13
SDS-PAGE of SUMO-APC/C gel filtration (GF) elution profile
B
50nm
Typical micrograph
C
2D classes
D
SUMOylated apoAPC/C with repositioned APC2WHB
Body 2 with repositioned APC2WHB after Multi Body refinement
SUMOylated apoAPC/C with density at APC2-APC4
Fourier Shell Correlation (FSC)
Resolution (1/Å)
0.143
3.99Å
3.78Å
3.78Å
E
Resolution (Å)
WHB domain with additional density
F
1
All good particles after 2D classification
1,002,696 particles
2
3D classification
16%
Bad particles
27%
Good particles
15%
Noisy particles
42%
Good particles
Refine
4
3
Mask
WHB/APC10/APC3
APC1 PC repeats
Bayesian polishing
3.49 Å
701,885 particles
Multiple rounds
Focused 3D classification without alignment after signal subtraction
12 iter.
20%
High occupancy
5
Revert to full particles and refine
3.78 Å
137,954 particles
Masks for
Multi Body Refinement
6
APC2WHB
3.78 Å
Body 2 with the best density

Figure S3. Biochemical and structural characterization of *in vitro* SUMOylated APC/C. Related to figure 3.

(A) Coomassie blue stained SDS-PAGE gel showing gel filtration (GF) fractions of *in vitro* SUMOylated APC/C.

(B) Representative cryo-electron micrograph.

(C) Typical 2D classes used for structure determination.

(D) Gold-standard Fourier Shell Correlation (FSC) curves of all SUMOylated APC/C reconstructions in this study.

(E) SUMOylated APC/C map colored by local resolution estimation.

(F) Three-dimensional classification scheme for SUMOylated APC/C structure determination, resulting in classes with $APC2^{WHB}$ domain density being clearly visible. All particles after 2D classification were refined together to give an initial model (**1**), which were 3D classified into four classes (**2**). The two best 3D classes were selected after visual examination, combined and refined together. Bayesian polishing was performed on these best particles with CTF refinement in RELION 3.0 (**3**). $APC2^{WHB}$, APC10, APC3 and APC1 PC repeats were then masked and the signal from the rest of the APC/C was subtracted, and focused 3D classification without alignment was performed to give three classes (**4**), only one of which had high occupancy of $APC2^{WHB}$ (**5**). Full particles with high $APC2^{WHB}$ occupancy were then refined and the APC/C was split into three bodies for multi body refinement (**6**), which gave a final map with a well-resolved $APC2^{WHB}$.

Figure S4

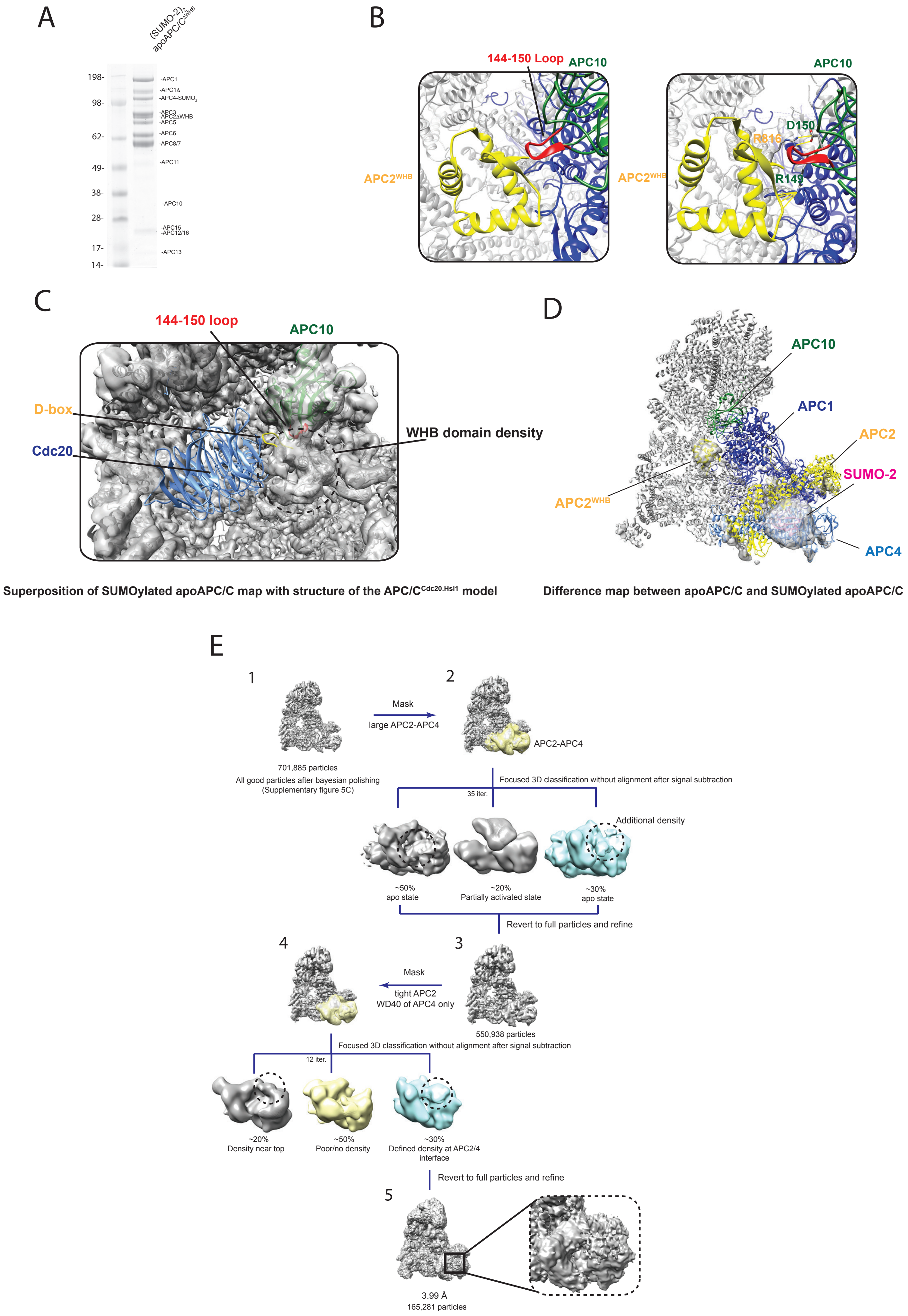

A
(SUMO-2)$_3$ apoAPC/C^{ΔWHB}
198-
98-
62-
49-
38-
28-
17-
14-
-APC1
-APC1Δ
-APC4-SUMO$_2$
-APC3
-APC2ΔWHB
-APC5
-APC6
-APC8/7
-APC11
-APC10
-APC15
-APC12/16
-APC13
B
144-150 Loop
APC10
APC2^{WHB}
APC10
D150
R816
R149
APC2^{WHB}
C
144-150 loop
APC10
D-box
Cdc20
WHB domain density
Superposition of SUMOylated apoAPC/C map with structure of the APC/C$^{Cdc20.Hsl1}$ model
D
APC10
APC1
APC2
SUMO-2
APC2^{WHB}
APC4
Difference map between apoAPC/C and SUMOylated apoAPC/C
E
1
2
Mask
large APC2-APC4
APC2-APC4
701,885 particles
All good particles after bayesian polishing
(Supplementary figure 5C)
Focused 3D classification without alignment after signal subtraction
35 iter.
Additional density
~50%
apo state
~20%
Partially activated state
~30%
apo state
Revert to full particles and refine
4
3
Mask
tight APC2
WD40 of APC4 only
550,938 particles
Focused 3D classification without alignment after signal subtraction
12 iter.
~20%
Density near top
~50%
Poor/no density
~30%
Defined density at APC2/4
interface
Revert to full particles and refine
5
3.99 Å
165,281 particles

Figure S4. Details of APC2^{WHB} domain interaction with the rest of the APC/C. Related to figure 3.

(A) Coomassie Blue stained SDS-PAGE gel of SUMOylated APC/C$^{\Delta WHB}$ that was used for structure determination.

(B) APC2^{WHB} binds directly to amino acids 140-150 (140-150) loop of APC10.

(C) Repositioned APC2^{WHB} domain has only modest overlap with Cdc20. Superposition of SUMOylated apoAPC/C map onto the APC/C$^{Cdc20.Hsl1}$ structure (PDB ID 5G04) visualized in Chimera. APC10 – forest green; blue – Cdc20; D-box of Hsl1 – yellow; 144-150 loop – red.

(D) Model of the SUMOylated APC/C with repositioned APC2^{WHB} domain is overlayed with the difference map between SUMOylated apoAPC/C and apoAPC/C. Clear positive differences are visible for SUMO-2, APC2^{WHB} (below APC10) and within APC2^{CTD} and cullin repeats.

(E) Three-dimensional classification scheme for SUMOylated APC/C structure determination, resulting in class with clear density at the APC2-APC4 interface, assigned to SUMO-2. The processing was continued after Bayesian polishing described and shown in Figure S3F (**1**). APC4 and APC2 were masked and the signal was subtracted from the rest of the APC/C and focused 3D classification without alignment was performed to give three classes (**2**). apoAPC/C (no density), partially activated APC/C were excluded and only class with additional density was taken for subsequent processing. APC/C was refined again (**3**) and a tight mask around APC4 WD40 domain and APC2 were used for another round of focused 3D classification without alignment (**4**). A single class with defined density at APC2-APC4 interface was taken and refined to give a final map (**5**).

Figure S5

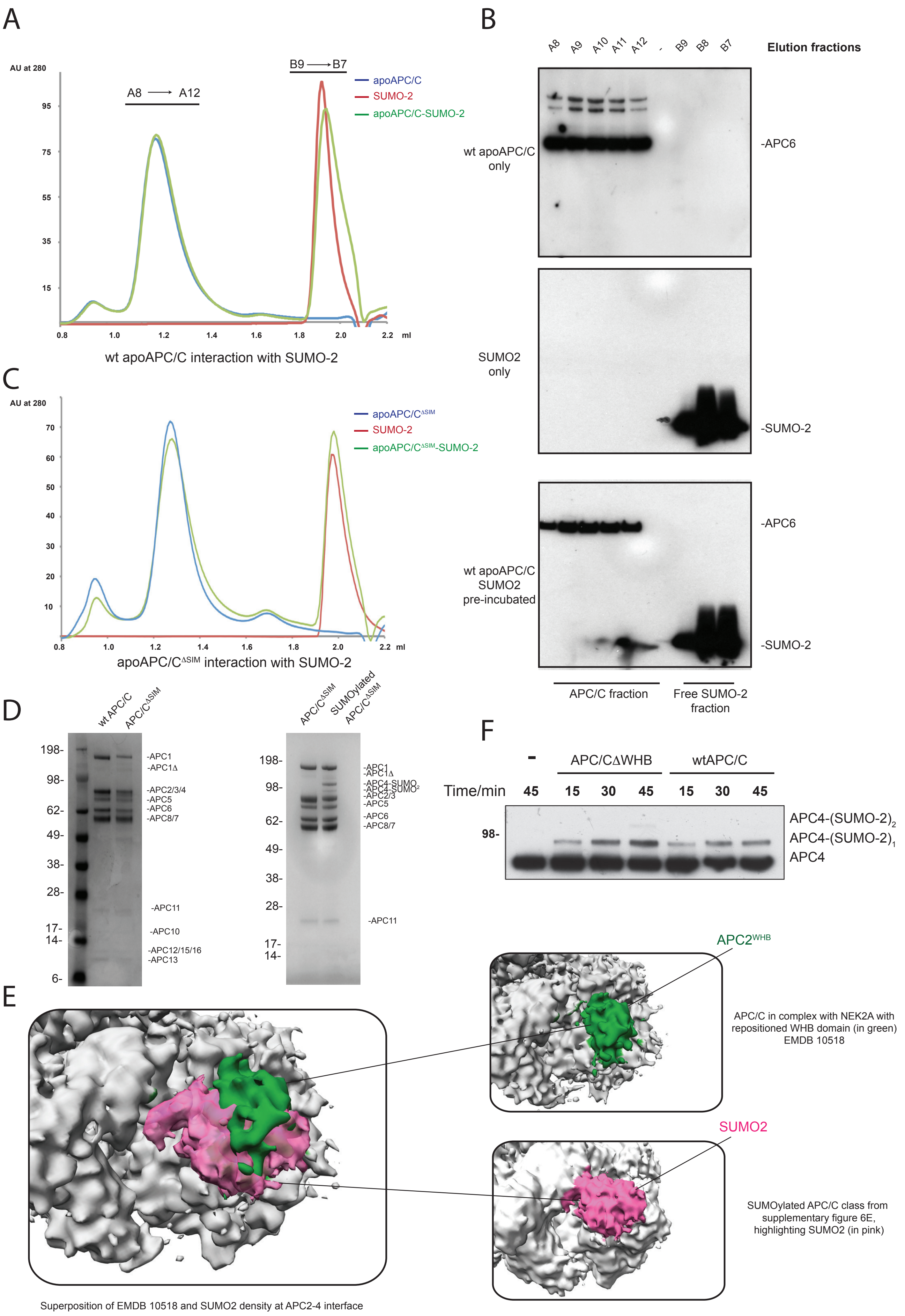
A
AU at 280
B9 → B7
A8 → A12
apoAPC/C
SUMO-2
apoAPC/C-SUMO-2
ml
wt apoAPC/C interaction with SUMO-2
C
AU at 280
apoAPC/CΔSIM
SUMO-2
apoAPC/CΔSIM-SUMO-2
apoAPC/CΔSIM interaction with SUMO-2
B
A8 A9 A10 A11 A12 - B9 B8 B7
Elution fractions
wt apoAPC/C only
-APC6
SUMO2 only
-SUMO-2
wt apoAPC/C SUMO2 pre-incubated
-APC6
-SUMO-2
APC/C fraction
Free SUMO-2 fraction
D
wt APC/C
APC/CΔSIM
-APC1
-APC1Δ
-APC2/3/4
-APC5
-APC6
-APC8/7
-APC11
-APC10
-APC12/15/16
-APC13
APC/CΔSIM
SUMOylated APC/CΔSIM
-APC1
-APC1Δ
-APC4-SUMO
-APC4-SUMO2
-APC2/3
-APC5
-APC6
-APC8/7
-APC11
F
APC/CΔWHB
wtAPC/C
Time/min
- 45
15 30 45
15 30 45
98-
APC4-(SUMO-2)2
APC4-(SUMO-2)1
APC4
E
APC2WHB
APC/C in complex with NEK2A with repositioned WHB domain (in green) EMDB 10518
SUMO2
SUMOylated APC/C class from supplementary figure 6E, highlighting SUMO2 (in pink)
Superposition of EMDB 10518 and SUMO2 density at APC2-4 interface

Figure S5. SUMO-2 directly binds to APC/C and APC/C$^{\Delta SIM}$. Related to figure 4.

(A) Analytical gel filtration chromatogram of SUMO-2 and APC/C, and SUMO-2 pre-incubated with the APC/C. APC/C at 1 μM was incubated with 60 μM SUMO-2 on ice for 30 min and then samples were run on a Superose 6 gel filtration column.

(B) Immunoblotting of peak fractions against SUMO-2 shows that SUMO-2 eluted in earlier fractions only when it was pre-incubated with the APC/C. The samples were analysed by immunoblotting against SUMO-2.

(C) Analytical gel filtration chromatogram of SUMO-2 and APC/C$^{\Delta SIM}$, and SUMO-2 pre-incubated with APC/C$^{\Delta SIM}$. APC/C$^{\Delta SIM}$ at 1 μM was incubated with 60 μM SUMO-2 on ice for 30 min and then samples were run on a Superose 6 gel filtration column.

(D) SDS PAGE gel of purified APC/C$^{\Delta SIM}$ compared to SUMOylated APC/C$^{\Delta SIM}$ which was used for structural work. The gel was stained with Coomassie blue.

(E) Comparison of the APC/C in complex with Nek2A (Alfieri et al., 2020), which exhibits APC2^{WHB} domain rearrangement to the APC2-4 interface, and SUMO2 density as observed in classes described in Figure S4E. EMDB 10518 was used to assess the APC/C-Nek2A complex; APC2^{WHB} is coloured green. SUMO2 is coloured pink.

(F) The SUMOylation reaction proceeded faster when the APC2^{WHB} was removed. SUMOylation reaction was performed as described in Methods.

Figure S6

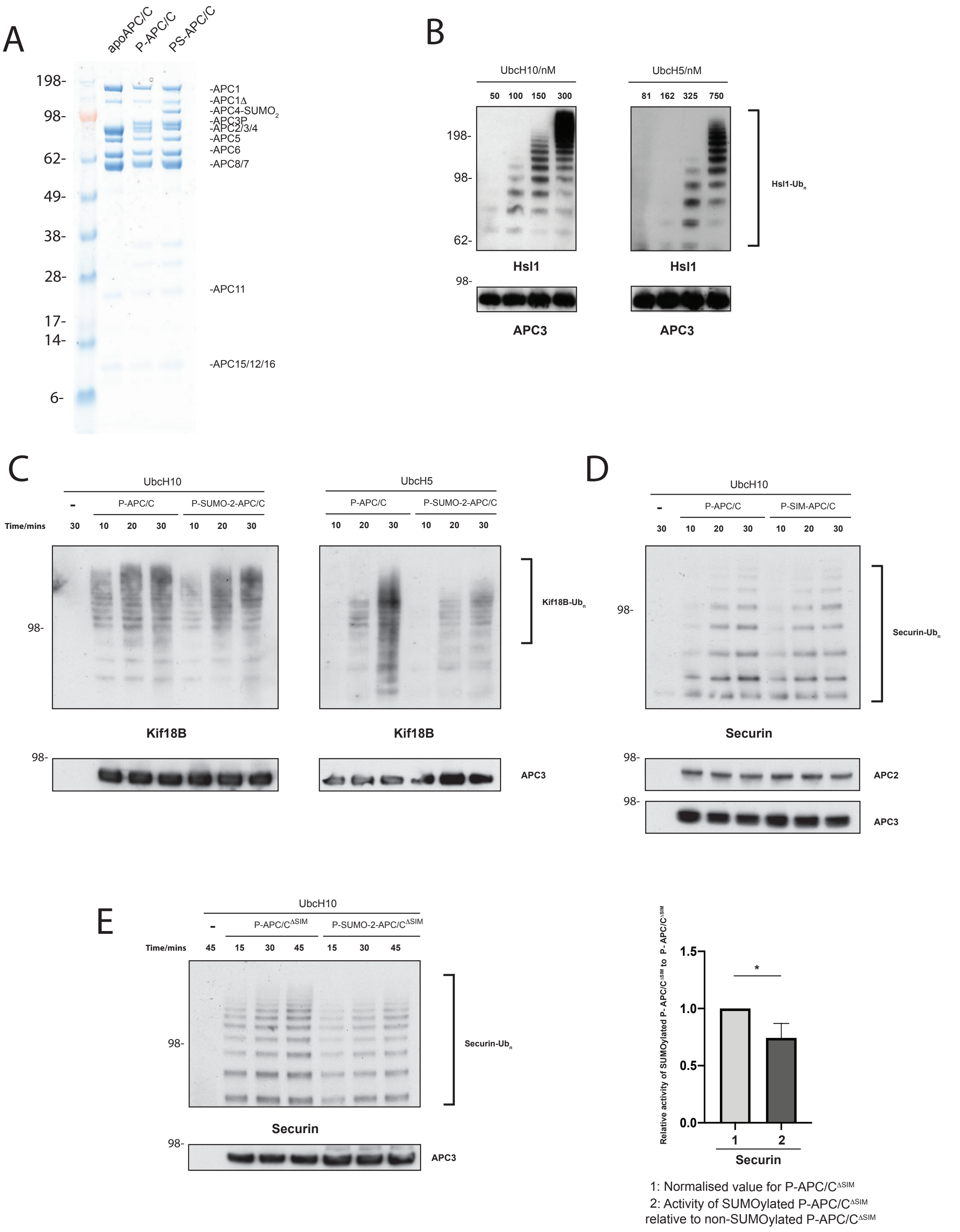
A
apoAPC/C
P-APC/C
PS-APC/C
198-
98-
62-
49-
38-
28-
17-
14-
6-
-APC1
-APC1Δ
-APC4-SUMO$_2$
-APC3P
-APC2/3/4
-APC5
-APC6
-APC8/7
-APC11
-APC15/12/16
B
UbcH10/nM
50 100 150 300
UbcH5/nM
81 162 325 750
198-
98-
62-
Hsl1-Ub$_n$
Hsl1
APC3
C
UbcH10
P-APC/C
P-SUMO-2-APC/C
UbcH5
Time/mins
30 10 20 30 10 20 30
Kif18B-Ub$_n$
Kif18B
APC3
D
P-SIM-APC/C
Securin-Ub$_n$
Securin
APC2
E
P-APC/C$^{\Delta SIM}$
P-SUMO-2-APC/C$^{\Delta SIM}$
45 15 30 45 15 30 45
Relative activity of SUMOylated P-APC/C$^{\Delta SIM}$ to P-APC/C$^{\Delta SIM}$
1.5
1.0
0.5
0.0
*
1
2
Securin
1: Normalised value for P-APC/C$^{\Delta SIM}$
2: Activity of SUMOylated P-APC/C$^{\Delta SIM}$ relative to non-SUMOylated P-APC/C$^{\Delta SIM}$

Figure S6. Ubiquitination assays. Related to figure 5.

(A) Coomassie stained SDS-PAGE gel showing different APC/C samples used in ubiquitination assays in Figure 5A, C, E.

(B) Titration of E2 enzymes used in the ubiquitination assay. The assays were performed for 10 min using Hsl1 as a substrate with indicated amounts of UbcH10 and UbcH5 without Ube2S enzymes. Ubiquitination was detected by immunoblotting against the His-tag of ubiquitin.

(C) Comparison of SUMOylated and non-SUMOylated APC/C ubiquitination activity towards Kif18B using UbcH10 as the sole E2 enzyme. The reactions were incubated for the time indicated in the Figure and ubiquitination was detected by immunoblotting against the His-tag of ubiquitin. The assays were performed at least in triplicate.

(D) Comparison of wild-type phosphorylated APC/C activity and phosphorylated APC/C$^{\Delta SIM}$ towards securin in the ubiquitination assay. UbcH10 was used as the sole E2 enzyme. Ubiquitination was detected by immunoblotting against securin. The assays were performed at least in triplicate.

(E) Comparison of SUMOylated and non-SUMOylated phosphorylated APC/C$^{\Delta SIM}$ ubiquitination activity towards securin using UbcH10 as the sole E2 enzyme. Ubiquitination was detected by immunoblotting against securin. The assays were performed at least in triplicate and quantified as described in Methods. The error bar represents mean with one standard deviation of the ratio of the SUMOylated/non-SUMOylated APC/C activity. One star indicates a significance smaller than 0.05. Significance was calculated using an unpaired Student's t-test.

Figure S7

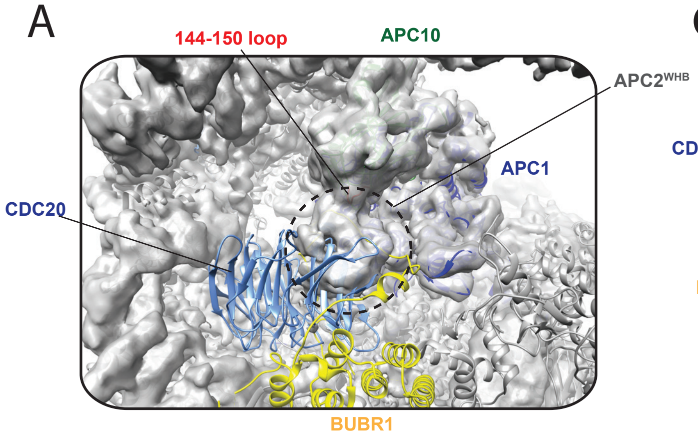

Superposition of SUMOylated apoAPC/C map with structure of the APC/C$^{MCC\text{-}Closed}$ complex in closed conformation

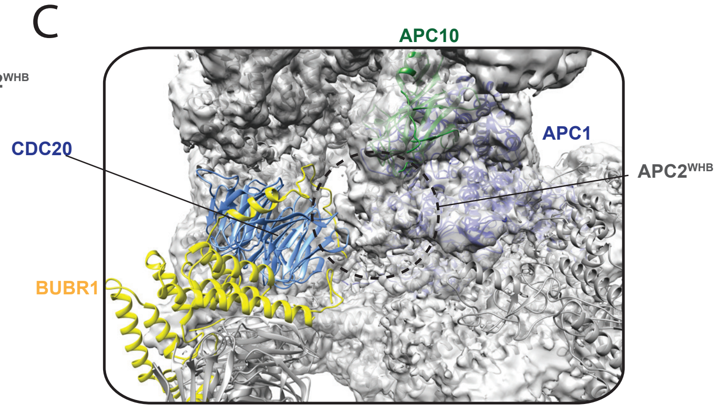

Superposition of SUMOylated apoAPC/C map with structure of the APC/C$^{MCC\text{-}Open}$ complex in open conformation

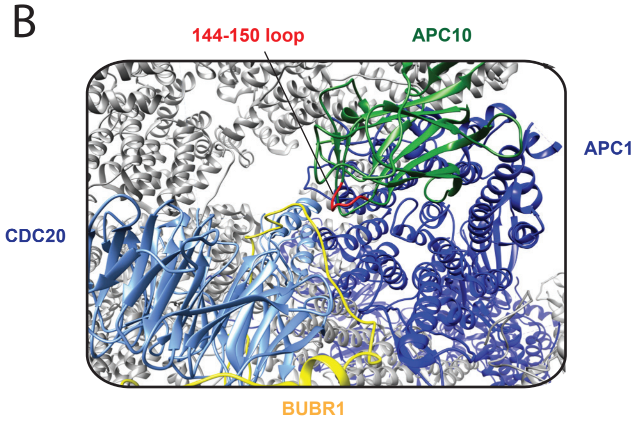

Structure of the APC/C$^{MCC\text{-}Closed}$ complex shows close contacts between BubR1 and 144-150 loop of APC10

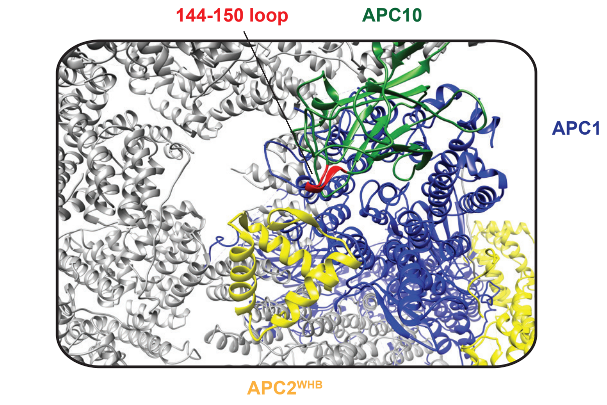

Structure of the SUMOylated apoAPC/C shows similar contacts between APC2^{WHB} and 144-150 loop of APC10

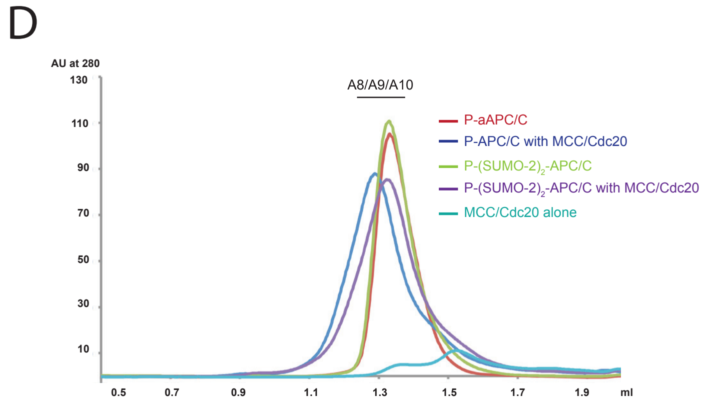

All absorption curves from MCC/Cdc20 reconstitution experiments plotted on the same coordinates

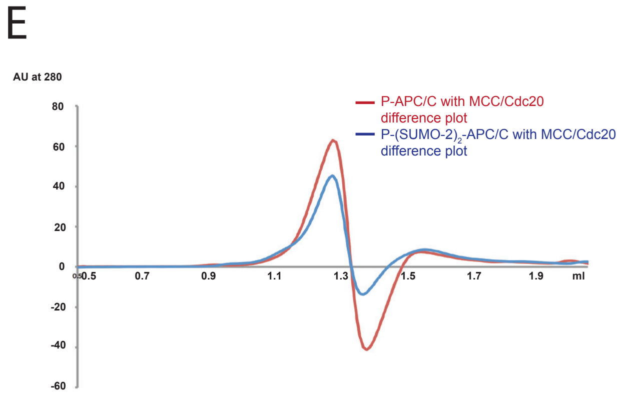

Absorption difference between APC/C and APC/C pre-incubated with MCC/Cdc20

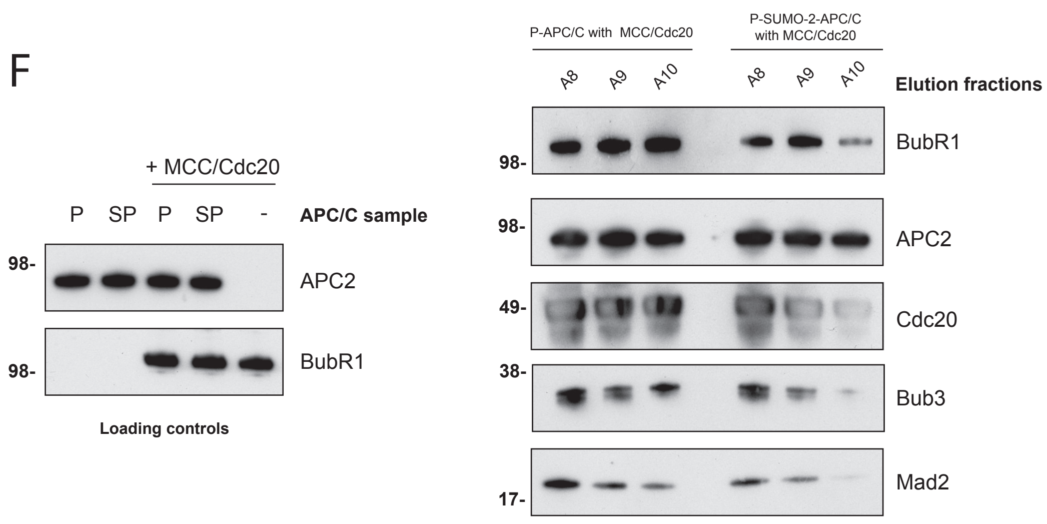

Figure S7. Comparison of SUMOylated apoAPC/C structure with MCC-APC/C^{Cdc20} structure. Related to figure 6.

(A) Superposition of the SUMOylated apoAPC/C map onto APC/C$^{MCC\text{-}Closed}$ structure (PDB ID 5LCW) shows a clear clash between APC2^{WHB} and BubR1 and the tilted Cdc20^{A}. APC1 – dark blue; Cdc20 – light blue; BubR1 – yellow, APC10 – forest green.

(B) BubR1 of the MCC and APC2^{WHB} bind to the same 144-150 loop of APC10. 144-150 loop of APC10 is coloured red.

(C) Superposition of the SUMOylated apoAPC/C map onto the MCC-APC/C$^{Cdc20\text{-}Open}$ structure (Alfieri et al., 2016) a minor clash between APC2^{WHB} and BubR1 or Cdc20^{A}. APC1 – dark blue; Cdc20 – light blue; BubR1 – yellow, APC10 – forest green.

(D) Chromatograms of all the samples plotted on the same coordinates without any modification. The fractions used for immunoblotting are indicated above the chromatograms.

(E) A plot of absorption difference at 280 nm between APC/C samples and APC/C samples pre-incubated with MCC and Cdc20. Chromatograms shown in Figure S7D were used to calculate the absorption differences.

(F) Left panel: immunoblotting against APC2 and BubR1, an MCC component, shows equal loading of all reactants for the reconstitution experiments, P: phosphorylated APC/C, SP: phosphorylated SUMOylated APC/C, **-**: sample without APC/C (i.e. MCC-Cdc20 alone). Right panel: immunoblotting against APC2 shows that equal amounts of APC/C are present in peak fractions, whereas immunoblotting against MCC components, BubR1, Bub3 and Mad2 shows reduced amount of MCC in the SUMOylated APC/C sample. The peak fractions taken for the immunoblotting are shown in Figure S7D.

**Data collection**	*In vitro* SUMOylated APC/C	
EM	FEI Titan Krios, 300 kV	
Detector	Falcon 3, counting mode	
Pixel size (Å)	1.07-1.085	
Magnification	75,000x	
Defocus range (μm)	2-3	
Electron exposure (e–/Å2)	0.5-0.6	
Number of micrographs	5,587	
**Reconstruction**	SUMOylated apoAPC/C with repositioned $APC2^{WHB}$	Body 2 with repositioned $APC2^{WHB}$ after Multi Body refinement
Particles used for final reconstruction	137,954	137,954
Software	Relion 3.0	Relion 3.0
Applied symmetry	C1	C1
Accuracy of rotations (degrees)	1.359	3.71
Accuracy of translations (pixels)	0.924	1.554
B-factor applied	-30	-30
Final resolution (Å)	3.78	3.78
FSC threshold	0.143	0.143
Reconstruction	SUMOylated apoAPC/C with density at APC2-APC4	
Particles used for final reconstruction	165,281	
Software	Relion 3.0	
Applied symmetry	C1	
Accuracy of rotations (degrees)	1.768	
Accuracy of translations (pixels)	1.368	
B-factor applied	-30	
Final resolution (Å)	3.99	
FSC threshold	0.143	

**Refinement**	SUMOylated apoAPC/C with repositioned $APC2^{WHB}$
**Initial model used**	5G05
**Map sharpening B factor (Å^2)**	-30
**Model composition**	
Chains	23
Non-hydrogen atoms	63827
Protein residues	8109
**B factors (Å^2)**	
Protein	135.53
**R.m.s. deviations**	
Bond lengths (Å)	0.005
Bond angles (°)	0.990
**Validation**	
MolProbity score	2.86
Clashscore	14.04
Rotamer outliers (%)	9.39
**Ramachandran plot**	
Favored (%)	92.66
Allowed (%)	7.13
Disallowed (%)	0.21

Table S1

Table S1. CryoEM data collection, processing information and refinement statistics. Related to Figure 3.
